# Post‐glacial range formation of temperate forest understorey herbs – Insights from a spatio‐temporally explicit modelling approach

**DOI:** 10.1111/geb.13677

**Published:** 2023-04-10

**Authors:** Wolfgang Willner, Johannes Wessely, Andreas Gattringer, Dietmar Moser, Eliška Záveská, Stefan Dullinger, Peter Schönswetter, Karl Hülber

**Affiliations:** ^1^ Department of Botany and Biodiversity Research University of Vienna Rennweg 14 Vienna 1030 Austria; ^2^ Vienna Doctoral School of Ecology and Evolution (VDSEE) University of Vienna Djerassiplatz 1 Vienna 1030 Austria; ^3^ Department of Botany University of Innsbruck Sternwartestr. 15 Innsbruck 6020 Austria; ^4^ Institute of Botany of the Czech Academy of Sciences Zámek 1 Průhonice 252 43 Czech Republic

**Keywords:** dispersal limitation, Europe, forest herbs, long‐distance dispersal, Pleistocene refugia, post‐glacial recolonization, range filling, spatio‐temporally explicit modelling

## Abstract

**Aim:**

Our knowledge of Pleistocene refugia and post‐glacial recolonization routes of forest understorey plants is still very limited. The geographical ranges of these species are often rather narrow and show highly idiosyncratic, often fragmented patterns indicating either narrow and species‐specific ecological tolerances or strong dispersal limitations. However, the relative roles of these factors are inherently difficult to disentangle.

**Location:**

Central and south‐eastern Europe.

**Time period:**

17,100 BP – present.

**Major taxa studied:**

Five understorey herbs of European beech forests: *Aposeris foetida*, *Cardamine trifolia*, *Euphorbia carniolica*, *Hacquetia epipactis* and *Helleborus niger*.

**Methods:**

We used spatio‐temporally explicit modelling to reconstruct the post‐glacial range dynamics of the five forest understorey herbs. We varied niche requirements, demographic rates and dispersal abilities across plausible ranges and simulated the spread of species from potential Pleistocene refugia identified by phylogeographical analyses. Then we identified the parameter settings allowing for the most accurate reconstruction of their current geographical ranges.

**Results:**

We found a largely homogenous pattern of optimal parameter settings among species. Broad ecological niches had to be combined with very low but non‐zero rates of long‐distance dispersal via chance events and low rates of seed dispersal over moderate distances by standard dispersal vectors. However, long‐distance dispersal events, although rare, led to high variation among replicated simulation runs.

**Main conclusions:**

Small and fragmented ranges of many forest understorey species are best explained by a combination of broad ecological niches and rare medium‐ and long‐distance dispersal events. Stochasticity is thus an important determinant of current species ranges, explaining the idiosyncratic distribution patterns of the study species despite strong similarities in refugia, ecological tolerances and dispersal abilities.

## 1 INTRODUCTION

Temperate forests are the dominant natural vegetation of large parts of Europe (Bohn & Neuhäusl, 2000; Leuschner & Ellenberg, 2017). However, during cold phases of the Pleistocene, forests were spatially restricted to small, climatically favourable areas. For most deciduous tree species, such refugial areas were located in southern Europe (Leroy & Arpe, 2007). Locations of refugia and post‐glacial recolonization routes have been proposed for numerous temperate trees based on a variety of data including molecular polymorphisms, pollen‐ and macrofossils as well as palaeo‐distribution modelling (e.g., Grivet & Petit, 2003; Magri et al., 2006; Petit et al., 2002; Svenning, Normand, & Kageyama, 2008). In contrast, our knowledge of the glacial refugia and post‐glacial migration of forest understorey herbs is still very limited. Phylogeographical studies (*Cyclamen purpurascens*: Slovák et al., 2012; *Hacquetia epipactis*: Urbaniak et al., 2018; *Helleborus niger*: Záveská et al., 2021) suggest that their glacial refugia were mostly congruent with those of their accompanying tree species. However, their current distribution ranges match those of dominant tree species to a varied degree and show a high level of idiosyncrasy in terms of geographical patterns.

The geographical distribution of any species is limited by a combination of environmental and dispersal constraints, with relative effect strengths varying across species (Soberón, 2007). For plants, climatic and soil conditions represent the most important environmental filters (Leuschner & Ellenberg, 2017; Pearson & Dawson, 2003). However, most forest herbs likely disperse poorly; that is, seeds are heavy and frequently have no appendages fostering regular wind or zoochorous dispersal (except for short‐distance ant dispersal via elaiosomes). Consistent with these morphological traits, various evidence suggests that the current distribution ranges of European forest understorey species bear signs of migration lags and incomplete range filling (Jiménez‐Alfaro et al., 2018; Svenning, Normand, & Skov, 2008; Willner et al., 2009). However, to what extent the highly idiosyncratic distribution patterns of individual species can actually be explained by dispersal processes has not been systematically evaluated yet. Moreover, interactions between dispersal and life‐history traits, such as age at maturity, flowering frequency, seed yield and clonal reproduction rate, are still poorly understood (Dullinger et al., 2015).

Beech forests are among the most widespread forest types in temperate Europe (Bohn & Neuhäusl, 2000), and harbour a characteristic set of understorey species (Willner et al., 2017). The range sizes of beech forest understorey species show a bimodal frequency distribution: most species are either widespread [i.e., they cover large parts of the range of European beech (*Fagus sylvatica*) or even extend beyond in some regions], or they have relatively narrow ranges, which are usually clustered around the glacial refugia of beech (Willner et al., 2009). Species ranges of the latter group are often characterized by disjunctions, that is, they consist of an archipelago‐like set of sub‐ranges. Such patchy distribution patterns may have emerged from a continuous wave‐like migration process at a slow to moderate rate and subsequent extinction of populations from intermediate areas, for example, due to changes of local conditions over time. Alternatively, they could have been the result of erratic long‐distance dispersal (LDD) events, creating regional distribution foci, combined with very slow further expansion from these foci (Jordano, 2017; Nathan, 2006). The disjunct range structures of the narrow‐range beech forest species hence represent an excellent model system for studying the possible interaction of ecological tolerances and dispersal processes in shaping the high variability in the current distribution patterns of forest understorey herbs.

**FIGURE 1:**
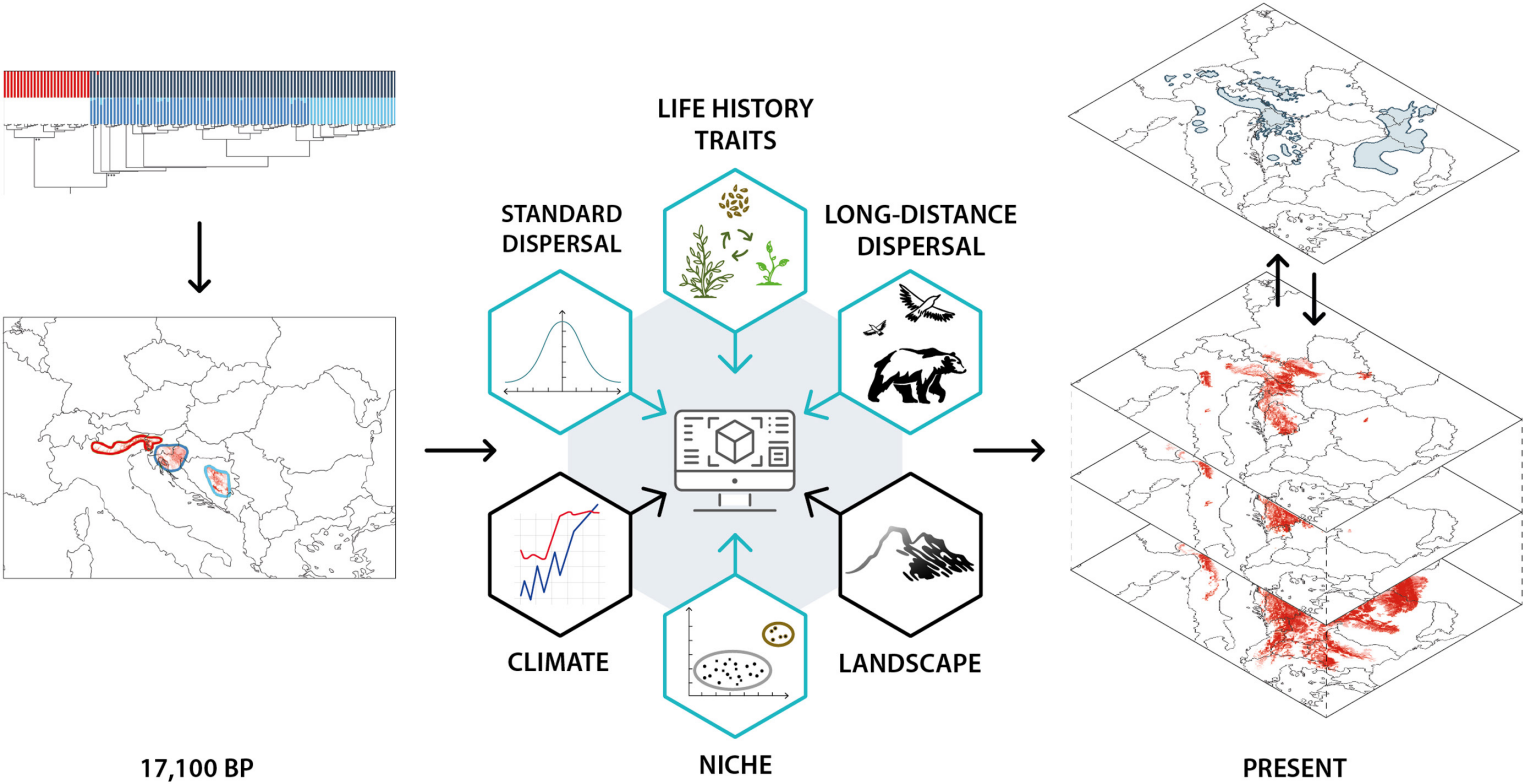
General workflow of our study. Spatio‐temporal dynamics of study species were modelled on a grid of 1 km × 1 km mesh size in annual time steps starting at 17,100 BP with the species occupying all climatically suitable and unglaciated cells inside the species’ glacial refugia, which were inferred from a phylogeographical analysis. Species‐specific parameter sets (blue hexagons), varied within plausible ranges in repeated simulation runs for each species, were combined with parameter sets of the study area (black hexagons), which were the same in all simulation runs. The model combined local species demography determined by life history traits with species distribution models (SDMs) defining the species’ niche and two types of dispersal among grid cells: a combination of exo‐ and endozoochoric dispersal representing the standard dispersal, as well as a vector‐independent long‐distance dispersal. Parameter settings were evaluated by comparing predicted current distribution ranges of the species to their mapped ranges.

In the present study, we simulate the post‐glacial range dynamics of five range‐restricted herbs typically found in the understorey of European beech forests using a dynamic, spatio‐temporally explicit model including niche, dispersal and demographic parameters (Figure 1). All simulations are based on phylogeographical reconstructions of the locations of glacial refugia of the species (Kirschner et al., submitted; Voisin et al., in prep; Záveská et al., 2021). As the true values of niche, dispersal and demographic parameters are largely unknown for the study species, we vary them within plausible ranges to answer the following questions: (a) Which combination of ecological tolerances, dispersal abilities and life‐history traits of each understorey herb allows the most accurate reconstruction of its current geographical range? In particular, do we need long‐distance dispersal for successful range reconstruction, and if yes, at which frequency? (b) Are the relative effects of these factors on post‐glacial range expansion similar among the study species or do the highly idiosyncratic distribution patterns of understorey herbs reflect strong species‐specific differences in the contribution of ecological tolerances and dispersal processes?

## 2 MATERIALS AND METHODS

### 2.1 Study species and study area

**FIGURE 2:**
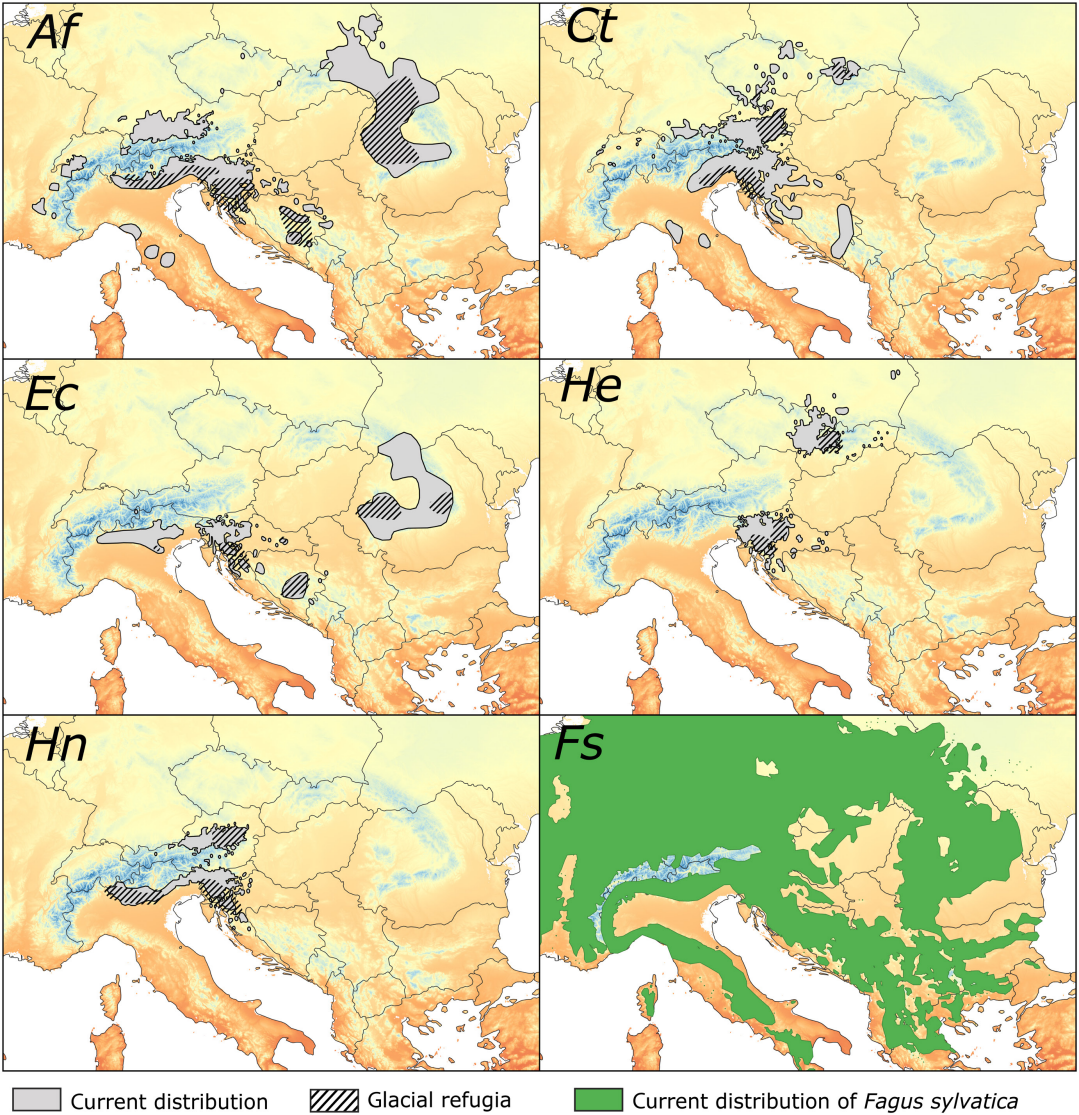
Current distribution (grey filled areas) and presumed glacial refugia (hatched areas) of five herbaceous forest understorey species, and the current distribution of European beech (green area). *Af*, *Ct*, *Ec*, *He*, *Hn* and *Fs* refer to the herbs *Aposeris foetida*, *Cardamine trifolia, Euphorbia carniolica*, *Hacquetia epipactis*, *Helleborus niger* and the deciduous tree *Fagus sylvatica*, respectively. Current distributions were obtained by intersecting published distribution maps with occurrence data from various sources (literature or herbarium specimens). Glacial refugia were inferred from phylogeographical data (Supporting Information Appendix S5). Background colours represent mean annual temperature ranging from −10 °C (dark blue) to 20 °C (dark orange).

As study species, we chose five herbaceous vascular plant species with a high fidelity for beech forests (according to Willner et al., 2009): *Aposeris foetida* (L.) Less. (Asteraceae), *Cardamine trifolia* L. (Brassicaceae), *Euphorbia carniolica* Jacq. (Euphorbiaceae), *Hacquetia epipactis* (Scop.) DC. (Apiaceae) and *Helleborus niger* L. (Ranunculaceae). All study species are perennial, entomophilous, have limited clonal reproduction and occur preferentially on calcareous bedrock (Fischer et al., 2008). We selected species from different families to cover a range of functional traits (see Supporting Information Appendix S1). Each study species has a highly idiosyncratic and disjunct range much smaller than that of European beech (Figure 2).

The study area (a rectangle with centroid 45° N, 17° E, extending 1,400 km in north–south and 2,110 km in west–east directions) covers large parts of the range of European beech in central and southeastern Europe. It is defined by the combined distribution ranges of the study species, which are restricted to the Alps, the Carpathians, the northern Apennines and the Balkan Peninsula (Figure 2) plus the intervening areas. Extensions towards south and north should cover potential distribution areas under historically colder as well as warmer climatic conditions, respectively. Except for most of the Alps, this area remained largely unglaciated during the Last Glacial Maximum (LGM; Ehlers et al., 2011) and overlaps with several putative refugia of temperate deciduous tree species (Magri et al., 2006). Beech forests are the dominant current vegetation on calcareous and siliceous bedrocks from the submontane to the upper montane belt. The climate of the study area is mainly continental‐temperate. Current mean annual temperature averaged over the study area is 9.7 °C (ranging from −9.9 to 19.2 °C), while mean annual temperature at the starting year of our simulation (17,100 BP) was 1.8 °C (from −13.2 to 14.3 °C) (see Section 2.3 for detailed information on the source of these climatic data).

### 2.2 Species distribution data, field sampling and inference of glacial refugia

We compiled published range maps of the study species from various distribution atlases and online databases (e.g., Switzerland: www.infoflora.ch; Italy: Poldini et al., 2002; Slovenia: Jogan et al., 2001; Croatia: https://hirc.botanic.hr/fcd; international: Meusel et al., 1978, 1992), unpublished databases (Austria: unpublished database of the project ‘Distribution Atlas of Vascular Plants of Austria’, https://plantbiogeography.univie.ac.at/research/distribution‐atlases/) as well as point occurrences from herbaria to obtain the current distributions as presented in Figure 2. Within these ranges, approximately regularly spaced populations of the study species were selected to collect leaf samples used to derive refugial areas (see next paragraph). Central coordinates of these sites were defined a priori and the presence of the study species within an area of 1 km × 1 km was evaluated in the field. We sampled material of *A. foetida, C. trifolia, E. carniolica, Hacquetia epipactis* and *Helleborus niger* at 123, 92, 77, 51 and 54 sites, respectively (Supporting Information Appendix S2).

Glacial refugia were inferred based on the analysis of restriction‐site associated DNA sequencing (RADseq) data following the strategy of Záveská et al. (2021). Specifically, we combined various parameters such as the occurrence of population‐ and region‐specific private alleles (indicating long‐term isolation), the geographical distribution of homogeneous gene pools identified by Bayesian clustering, and the relationships among populations and gene pools based on a maximum likelihood phylogeny. The results for *Helleborus niger* are fully published (Záveská et al., 2021); phylogeographical studies targeting the four other study species are underway (Kirschner et al., submitted; Voisin et al., in prep). We only included refugia with strong genetic support while areas where the phylogeographical data were inconclusive (central Balkan and northern Apennines for *C. trifolia*, western Alps for *A. foetida*) were excluded.

### 2.3 Environmental variables

Historic and current climatic conditions were derived from PaleoView (Fordham et al., 2017) and the Chelsa Climate database (Karger et al., 2017), respectively, and were combined using the delta‐method as described in Záveská et al. (2021). As starting point, we chose 17,100 BP, corresponding to a marked temperature minimum in the study area associated with Heinrich Stadial 1 (Hodell et al., 2017). Arithmetic means of climate variables (i.e., monthly precipitation as well as minimum, maximum and average monthly temperature) were averaged over 30‐year periods centred around each centenary from 17,100 to 0 BP (i.e., from 15,150 BC to 1950 AC) and used to calculate four bioclimatic variables representing amplitude and variability in temperature and precipitation. These are: annual mean temperature (bio1), temperature seasonality (bio4), annual precipitation sum (bio12) and precipitation seasonality (bio15). The bioclimatic variables were projected to a grid of 1 km × 1 km cell size covering the entire land surface within the study area at the respective time step (see below). As an additional environmental variable, we used topographic roughness measured as the standard deviation of the elevations of all 100 m × 100 m cells within each 1 km × 1 km land surface cell. The five environmental variables were checked for high correlations (Pearson's *r* > |.7|). An annual time series of each bioclimatic variable was derived from the centenary time steps via linear interpolation.

During the glacial periods, massive amounts of water accumulated in polar and continental ice sheets. The melting of these ice sheets caused a sea level rise of about 120 m to the current level (Lambeck et al., 2014). Lower sea levels opened migration corridors that are currently covered by the shallow waters of the Adriatic Sea. We used estimates of the sea level from Miller et al. (2005) and Lambeck et al. (2014) together with the 1 arc‐minute global relief model ETOPO1 (providing land topography and ocean bathymetry; Amante & Eakins, 2009) to reproduce the land surface extent at 100‐year time steps from 17,100 until 0 BP using ESRI arcgis 10.6. Climatic conditions for historic land surfaces, which are currently covered by sea – and hence lack climatic data in current data sources – were extrapolated from values along the current coastal regions applying a simple nearest neighbour allocation algorithm.

### 2.4 Simulations of spatio‐temporal range dynamics

CATS (Cellular automaton type model for simulating plant spread) is a spatially and temporally explicit model (Dullinger et al., 2012, 2015; Hülber et al., 2016; Wessely et al., 2017, 2022) operating on a two‐dimensional grid of variable mesh size (in this study 1 km). CATS combines simulations of local species demography with species distribution models (SDMs) by scaling demographic rates relative to occurrence probabilities predicted for the respective grid cell. Dispersal among grid cells is modelled as a combination of exo‐ and endozoochoric dispersal as well as by vector‐independent long‐distance dispersal. Time proceeds in annual steps. The annually changing occurrence probabilities at each grid cell (as projected by SDMs) affect the demography of local populations and thereby the number of seeds that are produced in each grid cell in the respective year. Consequently, local populations grow or decrease, become extinct or establish anew, and hence the species’ distribution across the study area changes as a function of the changing climate (Figure 1).

### 2.5 Modelling occurrence probability (SDMs)

We used SDMs to project the environmental suitability (i.e., the occurrence probability of species) across the study area for the five study species at annual time steps from 17,100 to 0 BP. These models relate species occurrences to the environmental variables described above at a resolution of 1 km. SDMs were based on generalized linear models (GLMs) using second‐order polynomials of all five environmental variables. In a first step, the coordinates of the sampling sites of each species were used as presences, while 10,000 pseudo‐absences were randomly drawn across Europe, but excluding the current range of the species, following recommendations by Barbet‐Massin et al. (2012) with minor modifications. To evaluate the plausibility of these models, which were based on the current climate only, we predicted the occurrence probability of the study species for two particularly cold periods, that is, at 17,100 and at 12,100 BP (the Younger Dryas). These predictions indicated clearly too narrow species niches, as there were either no suitable cells within the study area under past climatic conditions, or the suitable areas were far away from the refugia inferred from the phylogeographical analyses. Therefore, in a second step, the unglaciated cell with the highest occurrence probability (derived from the previous GLM) in each refugial area at 17,100 BP was selected, and the historic climatic conditions of this cell were used in addition to the current climatic conditions at the sampling sites to parameterize a new GLM. Two sets of pseudo‐absences were generated, one for 17,100 BP (excluding refugia and glaciated areas) and one for today (excluding the current distribution of the species), with 5,000 absences each, and they were assigned the historic (17,100 BP) or the current climate, respectively. This refitted GLM was used to predict annual occurrence probabilities. To account for stochasticity in drawing pseudo‐absences, the procedure was repeated 10 times and the resulting GLMs were averaged. The threshold for translating the predicted occurrence probabilities into presence or absence of each species at a site (further referred to as occurrence threshold, OT) was defined such that it optimized the true skills statistic (TSS), a measure of predictive accuracy derived from comparing observed and predicted presence–absence maps (Allouche et al., 2006). Glaciated areas were defined as in Ehlers et al. (2011) supplemented by Zasadni and Kłapyta (2014) for the Tatra Mountains. Ice‐free islands within the closed ice shield of the Alps were disregarded, as nunatak survival can be ruled out for beech forest understorey species.

If the basic assumption that narrow‐range beech understorey species have dispersal‐limited current distribution ranges is true, fitting SDMs with these current distributions may underestimate the species' niche breadth (Early & Sax, 2014). In addition, evolutionary or phenotypic adaptation may have modulated the niches of populations over the past 17,100 years. Current ecological tolerances might thus not fully integrate the species' ecological potential over time. For these reasons, we modulated the species' fitted ecological niches in our simulations in the following way. Each predictor term in a GLM regression formula represents one dimension of the multidimensional niche (Hutchinson, 1957) by a parabolic relationship between occurrence probability and predictor value. To determine niche breadth, these continuous functions need to be truncated. To do so, we projected occurrence probabilities along each predictor gradient separately (keeping all other predictors constant at the species' optimum value), and defined niche breadth as the interval between the upper and lower predictor values at which the probability dropped below OT. To extend niche breadth, one limit of each niche dimension (either the upper or the lower one) was shifted towards climatic conditions dominant during the glacial period. Thus, limits of bio1, bio4, bio12 and bio15 were extended towards lower annual mean temperatures, higher temperature seasonality, lower annual precipitation and higher precipitation seasonality, respectively. We then adjusted the regression coefficients in such a way that the regression passes through the OT at the shifted niche limit and the unchanged opposite limit by solving the resulting system of linear equations using mathematica (version 12.0; Wolfram Research, 2019). For our simulations, we used three levels of niche breadth extension (NBE): 0% (representing the data‐derived niche without extension), 10% and 20%. This procedure of niche extensions prevents the application of SDM modelling techniques apart from GLMs. A schematic illustration of such niche extension is given in Supporting Information Figure S3.

### 2.6 Demographic modelling

Environmental dependence of local demography of each study species was modelled by linking demographic rates (seed persistence, germination, survival, fecundity and clonal reproduction) to occurrence probabilities predicted by SDMs by means of sigmoid functions (Dullinger et al., 2012). Rate values were bounded between zero and the species‐specific maximum value of the respective rate derived from literature and databases (Supporting Information Appendix S1). The sigmoidal curve was calibrated by defining the rates’ values at the OT, such that the size of an isolated population remained stable in time (see Hülber et al., 2016, for details). In addition, survival, germination and clonal reproduction were modelled as density‐dependent processes using the species’ local carrying capacity as the upper threshold (Hülber et al., 2016). Carrying capacity was defined as the maximum number of adult individuals of a species per site, and it was linked to occurrence probabilities in the same way as demographic rates (Supporting Information Appendix S1). To account for uncertainty in parameters of demographic rates, we assigned each species two sets of values representing the upper and lower end of a plausible range of values. Demographic processes were also subjected to stochastic fluctuations (Dullinger et al., 2012). Therefore, small populations may go extinct even if the environmental conditions are suitable.

### 2.7 Dispersal modelling

Seeds were dispersed via three dispersal modes. First, most of the annual seed yield remained within the 1 km × 1 km cell of origin (further referred to as ‘local dispersal’ or LOD). Second, two kernels representing exo‐ and endozoochorous vectors were fitted based on species‐specific morphological traits of the seeds, representing the regular dispersal up to a few kilometres distance (‘standard zoochorous dispersal’, SZD). These kernels are based on 10,000 simulated random walks of a ‘general large mammalian seed dispersal vector’ (Dullinger et al., 2015) for seeds of each of the five study species. Kernels were derived as empirical density functions of these distances between a random seed uptake and the points of seed defecation or dropping from fur. Time until seed detachment depended on seed mass and seed shape following Römermann et al. (2005). We assumed that, on average, the same amount of seeds was successfully dispersed by exo‐ and endozoochory. However, it should be noted that a considerable amount of seeds dispersed by SZD is deposited in the cell of origin (on average between 35 and 42% depending on the species), thus effectively adding to LOD. Detailed kernels for each species are given in Supporting Information Figure S4. We did not use anemochorous kernels because seeds of the study species are transported over just a few metres by wind (Dullinger et al., 2015).

As a third dispersal mode, random dispersal events covering much longer distances (ranging several to hundreds of kilometres) were simulated independent of seed traits. They represent rare dispersal events, which are considered particularly important for the post‐glacial migration of temperate forest understorey herbs (Cain et al., 1998). Long‐distance dispersal (LDD) was simulated by distributing a certain amount of seeds randomly within a square of species‐specific side length and centred in the source cell. The square dimension was derived based on results of continuous phylogeographical diffusion analysis using relaxed random walks (Lemey et al., 2010) of the collected genetic data (Supporting Information Appendix S5). Side lengths of the squares were 995 km (*A. foetida*), 805 km (*C. trifolia*), 791 km (*E. carniolica*), 793 km (*Hacquetia epipactis*) and 761 km (*Helleborus niger*).

### 2.8 Simulation set up and simulation initialization

We ran CATS for each study species under a full factorial combination of the two demographic settings, three niche breadths, five levels of SZD and four levels of LDD. We assumed the proportion of seeds being dispersed from each occupied cell via LDD to be 0, 10^−7^, 10^−6^ and 10^−5^ of the overall seed yield of this cell, respectively. We further assumed the proportion of SZD to be 5∙10^−8^, 5∙10^−7^, 5∙10^−6^, 5∙10^−5^ and 5∙10^−4^ of the remaining seed yield. The rest of the seeds remained in the source cell (LOD). Lower SZD values were not included in the final simulations because they had the effect that the species showed no range expansion from refugia (and locations reached by LDD). To consider stochastic elements in the model (see Dullinger et al., 2012), each setting was replicated 10 times, resulting in 1,200 simulation runs per species. All simulation runs started at 17,100 BP with species‐specific initial distributions, defined as all unglaciated cells inside the species’ glacial refugia. Initial population sizes were equivalent to the carrying capacity. Populations in cells with an occurrence probability < OT (i.e., unsuitable cells) produced no seeds. Furthermore, populations below the lowest occurrence probability of all observed populations were halved each year (i.e., they went extinct).

### 2.9 Evaluation of model results

The mapped (i.e., derived from literature and online sources) and predicted current distribution ranges (i.e., representing model results) of the five species were compared at 50 km × 50 km resolution, which corresponds to the approximate accuracy of the former. A 50 km × 50 km grid cell was considered as being currently occupied by the species if any part overlapped with the mapped distribution range (Figure 2), while it was considered as being predicted to be occupied if it contained at least one 1 km × 1 km cell predicted to be occupied by the simulation run. Subsequently, we calculated the proportion of accurately predicted presences and absences (i.e., sensitivity and specificity) and the true skills statistic (TSS, i.e., sensitivity + specificity – 1) (Allouche et al., 2006). The TSS value indicates model quality and is used as a measure of the match between the predicted current distribution derived from the CATS simulation and the mapped current distribution of a species (i.e., the higher the TSS the better the match). To test whether optimal parameter settings were sensitive to the number of model replicates, we randomly draw five to nine replicates (from the 10 replicates available) and compared the results with those based on the full set of replicates. We applied 100 random selections for each sample size (Supporting Information Appendix S6).

## 3 RESULTS

Continuous phylogeographical diffusion analyses (Supporting Information Appendix S5) identified the north‐western Balkan Peninsula as a refugium for all five species, supplemented by further species‐specific refugia in the Carpathians, the central Dinaric Alps and/or the southern and north‐eastern Alps (Figure 2).

**FIGURE 3:**
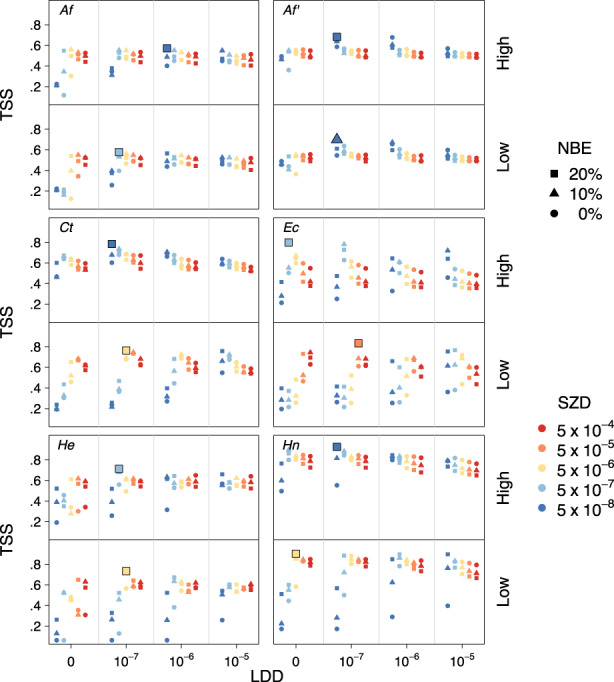
Match between the mapped current distribution of species and the current distribution predicted by model runs varying in the parameter settings for niche breadth extension (NBE), standard zoochorous dispersal (SZD) and long‐distance dispersal (LDD) evaluated as the true skills statistic (TSS). Demographic rates were assumed to be at the upper (‘high’) and lower (‘low’) ends of a plausible range of values. Best parameter settings are indicated with a larger symbol. Shown are the model runs with the highest TSS value out of 10 replicates with the same parameter setting for each species. *Af*, *Aposeris foetida*; *Af'*, *Aposeris foetida* evaluated within a restricted area excluding the Carpathians (see Figure 4); *Ct*, *Cardamine trifolia*; *Ec*, *Euphorbia carniolica*; *He*, *Hacquetia epipactis*; *Hn*, *Helleborus niger*.

**FIGURE 4:**
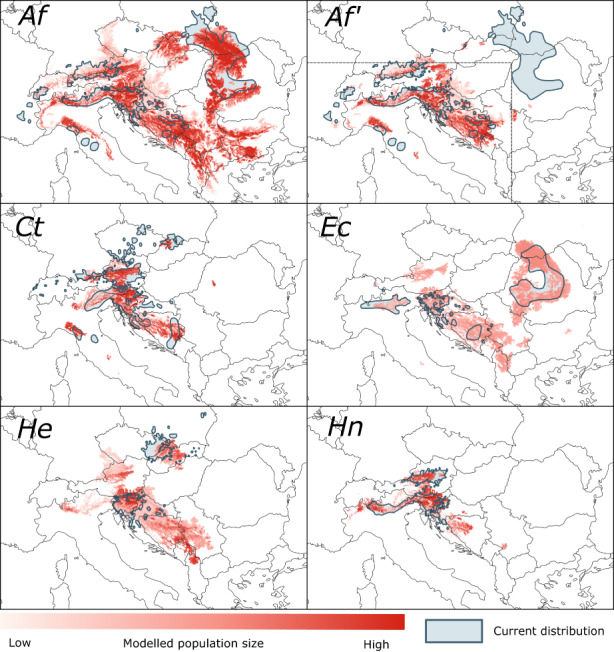
Comparison of the mapped current distributions of species (blue polygons) and the current distribution predicted by the model runs with the highest match (red). Colour intensity indicates the modelled population size in a 1 km × 1 km cell (dark red representing maximum population size according to the species‐specific carrying capacity). *Af*, *Aposeris foetida*; *Af'*, *Aposeris foetida* evaluated within a restricted area excluding the Carpathians (dashed line); *Ct*, *Cardamine trifolia*; *Ec*, *Euphorbia carniolica*; *He*, *Hacquetia epipactis*; *Hn*, *Helleborus niger*. For complete simulations, see Supporting Information Videos S7–S12.

**FIGURE 5:**
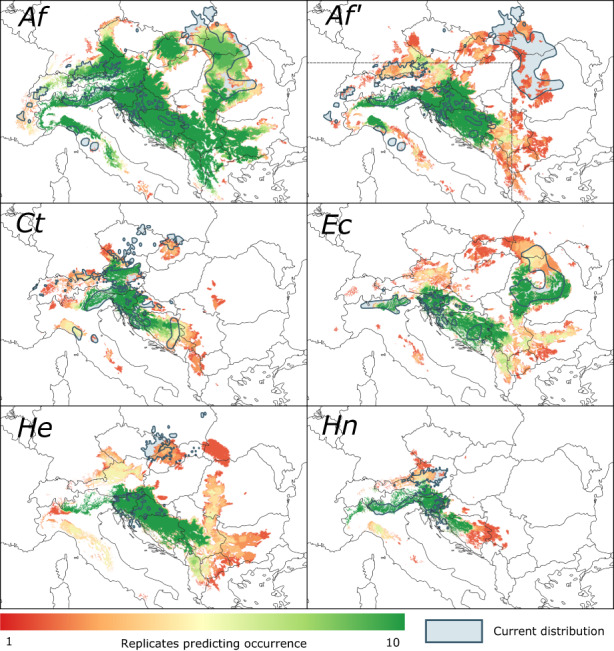
Areas predicted to be occupied by 10 replicated model runs using the parameter setting that includes the highest match with the mapped current distribution of the study species. Colours indicate the number of replicates in which a 1 km × 1 km cell was occupied, ranging from 1 (dark red) to 10 (dark green). Current species’ ranges are shown as blue polygons. *Af*, *Aposeris foetida*; *Af'*, *Aposeris foetida* evaluated within a restricted area excluding the Carpathians (dashed line); *Ct*, *Cardamine trifolia*; *Ec*, *Euphorbia carniolica*; *He*, *Hacquetia epipactis*; *Hn*, *Helleborus niger*.

Predicted current distributions derived from the CATS simulation, and thus their match with the mapped current distributions of species, varied vastly among the various settings of the parameters NBE, demography, SZD and LDD (Figure 3). Settings with the strongest match were obtained with broad niches (NBE = 20%) and a low, but non‐zero proportion of seeds dispersed by LDD (10^−7^) (Table 1, Figure 4, Supporting Information Videos S7–S12). However, predicted current distribution ranges varied strongly among replicates of the same settings, especially if SZD was low and LDD was > 0 (Figure 5 and Supporting Information Figure S13). Under higher LDD rates, species colonized all climatically suitable areas and, hence, covered areas much bigger than their mapped current distribution. In contrast, zero LDD in combination with low SZD did not allow species to expand substantially beyond their glacial refugia (Supporting Information Figures S14–S23).

**TABLE 1:** Parameter settings revealing the best match between predicted and mapped current distributions of the five study species.

Species	NBE (%)	SZD	LDD	Demography
*Aposeris foetida*	20	5∙× 10^−7^	10^−7^	Low
*A. foetida* in restricted area	20	5∙× 10^−8^	10^−7^	High
*Cardamine trifolia*	20	5∙× 10^−8^	10^−7^	High
*Euphorbia carniolica*	20	5∙× 10^−5^	10^−7^	Low
*Hacquetia epipactis*	20	5∙× 10^−6^	10^−7^	Low
*Helleborus niger*	20	5∙× 10^−8^	10^−7^	High

*Note*: Demography ‘low’ and ‘high’ refer to two sets of values representing the upper and lower ends, respectively, of a plausible range of values for seed persistence, germination, survival, fecundity and clonal reproduction. *Aposeris foetida* was evaluated both across the entire study area and within a restricted area excluding the Carpathians.

Abbreviations: LDD, long‐distance dispersal; NBE, niche breadth extension; SZD, standard zoochorous dispersal.

In contrast to NBE and LDD, SZD differed strongly among the best parameter settings (Table 1). Thereby, demography and SZD turned out to be antagonistic, with best matches reached either when SZD was low (i.e., 5∙10^−7^ or 5∙10^−8^) and demographic values were high, or when SZD was intermediate (about 5∙10^−6^) and demographic values were low (Figure 3). Higher SZD values resulted in strong over‐predictions of species’ ranges (Supporting Information Figures S14–S23).

All five species survived in each of the 1,200 simulation runs. However, populations of some refugial areas went extinct immediately after the start of the simulation under all settings, even though two of these refugia (*A. foetida* in the Carpathians and *H. niger* in the north‐eastern Alps) are strongly supported by the phylogeographical findings. Indeed, best matches between predicted and mapped current distribution ranges of *A. foetida* were considerably higher when the Carpathians were excluded from the area of evaluation (Table 1, Figure 4).

Supporting Information Videos S24–S43 showing the recolonization history of all five species from 17,100 to 0 BP in a spatio‐temporally explicit way under various settings are provided at https://phaidra.univie.ac.at/o:1429623.

## 4 DISCUSSION

We found a surprisingly consistent pattern of parameter settings for the best simulation runs among the five study species (Table 1). All of them included the highest niche breadth extension (of 20%). Although including climates of LGM refugia, niches without additional extensions were too narrow to allow the reconstruction of post‐glacial range formation of all five species. Moreover, the current distribution pattern of the study species could only be successfully predicted if we assumed strong dispersal limitation, with only small proportions of seeds being dispersed over distances beyond 500 m (i.e., outside of the 1 km^2^ cell, SZD with max. 10^−5^). These two results are not independent from each other because simulations using high dispersal rates performed even worse when including wider niches (Figure 3). At the same time, our results underline that a small fraction of the seeds had to move dozens to even hundreds of kilometres (LDD of 10^−7^) to generate the ranges that we see today. These findings are consistent with the fat‐tailed dispersal kernels postulated for explaining post‐glacial tree migration rates in North America (Clark, 1998). Taken together, our results support the hypothesis that the formation of current ranges in all five understorey species was driven by a few successful dispersal events over very large distances, followed, or paralleled to a certain extent, by slow and likely yet incomplete filling of the suitable area in between.

In accordance with earlier studies (e.g., Early & Sax, 2014) our results suggest that the ecological potential, or fundamental full niche breadth, is difficult to derive from current distribution data, especially in the case of narrow‐range, and thus often dispersal‐limited, species such as the ones we focused on. When simulating biogeographical trajectories over longer time spans, two factors may additionally constrain successful reconstruction. The first one is niche evolution through time. In fact, former populations of the same species may have differed from those of today in their ecological tolerances, allowing them to colonize and migrate through areas that nowadays appear outside the species’ niche. The likelihood, frequency and ecological magnitude of such niche change is unknown, but both data (Lavergne et al., 2010; Razgour et al., 2019) and models (Cotto et al., 2017; Wessely et al., 2022) suggest that climatic niches can evolve on centennial scales. Second, the climate of the past might not have present‐day analogues, at least within the species’ current range. In particular, there may be combinations of climatic variables that are no longer realized today. Hindcasting based on current distribution patterns is at best uncertain under these circumstances. The fact that our simulations predicted *A. foetida* and *Helleborus niger* to go extinct in regions proven as refugia by molecular data might be due to past combinations of cold, dry and continental conditions that do not exist anymore within our study area today.

We used a spatial resolution of 1 km × 1 km for our model implying a compromise between technical capabilities (concerning data availability and computer power) and the ecological requirements to obtain solid results. On the one hand, the application of SDMs to past environments requires reliable data on the historic climatic conditions (Svenning et al., 2011). While palaeoclimatic models have made substantial progress during the last few decades, regional projections are still afflicted by many uncertainties (Harrison et al., 2015). In particular, there is a discrepancy in the spatial resolution of the global climate models and our SDMs. The former have a resolution of 3° (*c*. 220 km × 330 km; Fordham et al., 2017) and were downscaled to a resolution of 1 km × 1 km, assuming the current fine scale pattern of climatic conditions to be constant over time. On the other hand, such a resolution does not capture microclimatic peculiarities, which might be particularly important in mountainous landscapes, which prevail across considerable parts of our study area. However, while our approach might be insensitive to local extinctions driven by environmental variation (or stochastic demographic processes) at spatial scales finer than 1 km^2^, it seems highly unlikely that such local extinction events could lead to large unoccupied regions within the species’ ranges. On the contrary, our approach might even underestimate species persistence as microrefugia with locally suitable conditions might potentially sustain populations even in cells that were predicted as unsuitable (Stark & Fridley, 2022). Indeed, microrefugia are a potential further explanation for our failure to reconstruct persistent populations in some refugial areas. Topographic roughness, the variable we used to consider the diversity of potential microsites within a cell, improved our models considerably as comparison with climate‐only models demonstrated (data not shown).

Despite the coarse resolution of climatic data, we are confident that the (currently) realized niche of each of our study species is well captured by the models, that is, that the niche breadth realized at 1 km^2^ at the continental scale correctly reflects the one realized at a finer scale within a more limited geographical extent. This does not preclude that our projections may underestimate the distributions of the species because microsites with suitable conditions within an otherwise unsuitable 1 km^2^ cell are not appropriately captured (see above). However, we assume that for the underestimation of the niches themselves (not the distributions) post‐glacial dispersal limitation is more relevant (Early & Sax, 2014). Indeed, naturalized occurrences of the study species outside their current native ranges (e.g., in Great Britain and Scandinavia) as available at the Global Biodiversity Information Facility (GBIF, http://www.gbif.org) suggest that the fundamental niches of the species are actually broader than the currently realized ones, allowing them to persist under climatic conditions with which they are currently not confronted.

Apart from microclimatic variation within cells, topographic roughness might serve as a proxy for soil properties that we could not account for due to the lack of sufficiently fine‐scaled maps. Beech forests are known to avoid moist soils (Leuschner & Ellenberg, 2017), and flat regions with extensive areas of moist soils might pose migration barriers for species adapted to well‐drained slopes, even if they were climatically suitable. In addition, the calcium content of the bedrock, another important determinant of the distribution of beech forest species (Ujházyová et al., 2016), was not covered by our models. Large areas with acidic bedrock, as found for instance at the south‐eastern margin of the Alps, might have been strong barriers for the northward migration of basiphilous forest herbs. Thus, migration rates in our simulations might be overestimated in the respective parts of the study area.

Earlier studies had already argued that current ranges of many understorey plants cannot be explained via the ‘standard’ or ‘usual’ dispersal processes alone (Cain et al., 1998; Griffin & Barrett, 2004). However, what is a standard and a non‐standard dispersal event is not formally defined and often interpreted differently by individual authors. As an example, many temperate forest understorey herbs produce elaiosomes (Howe & Smallwood, 1982). Their ‘standard’ dispersal vector is hence ants. However, it is well established that larger herbivores can and actually do transport seeds of ant‐dispersed species in their fur or guts (Vellend et al., 2003). While successful dispersal by these means may be considerably less likely, their frequency might still qualify them as a ‘standard’ dispersal means (see Green et al., 2022), with an at least roughly quantifiable potential to transport seeds by several kilometres. We therefore consider these processes as standard zoochoric dispersal (SZD). Finally, there are dispersal events that have very unusual mechanisms, including unusual behaviour of standard vectors (e.g., animal migration over large distances within a short time) and secondary dispersal (e.g., by carnivores that have predated on herbivores) (Green et al., 2022; Higgins et al., 2003; Nathan et al., 2008). These vectors have the potential to carry seeds over dozens or even hundreds of kilometres.

Our simulations suggest that all three of these dispersal processes likely contributed to the range formation in our study species. While the vast majority of seeds is dispersed locally, and mainly serves long‐term in‐situ survival of the species, actual range expansion is driven by an interaction of rare medium‐distance (SZD) and non‐standard, long‐distance dispersal events. Thereby, SZD drives slow expansion of existing populations, nevertheless with a considerably higher rate than pure ant dispersal would allow for (Cain et al., 1998). This process is probably responsible, in the first place, for the current sizes of subranges, that is, coherent patches within the disjunct current ranges. The LDD events, by contrast, were most likely responsible for the establishment of new patches further away from glacial refugia.

SZD and the local demography of species seem to be two sides of the same coin as high SZD combined with low demographic rates leads to similar results as low SZD combined with high demographic rates (Figure 3). Presumably, the crucial parameter is the number of dispersed seeds, which can be raised either by increasing the proportion of seeds being dispersed or by boosting the seed yield of a population. However, LDD introduces a strong stochastic element into range dynamics. In our model, LDD is determined only by its frequency, but acts as a chance event in terms of the distance and direction of dispersal. As a consequence, the same parameter setting can result in some runs closely resembling the real distribution of the species, while others show marked deviations due to the colonization of actually unpopulated areas or the failure to establish populations after the LDD event. Indeed, we defined the best parameter setting based on the simulation that delivered the best spatial match between predicted and current species distributions. However, across the 10 replicate simulations this ‘best’ parameter setting often produced predictions that were less well correlated with the current species' range than single runs with other settings, both with respect to the size of the predicted range and its spatial configuration (Figure 5 and Supporting Information Figure S13). Using a higher number of replicates might improve the match between predicted and mapped current distributions, but likely without changing the best parameter settings (Supporting Information Appendix S6).

Our results thus add a new spotlight to the discussion of driving forces of post‐glacial range formation of forest understorey herbs. In addition to the ecological niche and limited dispersal, current species ranges also seem to be the product of stochastic and unpredictable dispersal events. In this perspective, species’ abilities to overcome migration barriers during the recolonization of central Europe from Pleistocene refugia (e.g., the main chain of the Alps or the Pannonian Plain, both not inhabitable for most beech forest understorey species) depended on erratic long‐distance dispersal events, which might have happened at some time in the past or still have not yet happened. This interpretation explains the highly idiosyncratic patterns of the study species’ ranges (Figure 2), and also the markedly different phylogeographies reconstructed with genomic and molecular approaches (Slovák et al., 2012; Záveská et al., 2021). Of course, there are also many understorey herbs with huge, continuous ranges largely resembling that of beech or even surpassing it in some parts of Eurasia, especially in the north‐east (e.g., *Galium odoratum, Mercurialis perennis*, *Sanicula europaea*; Meusel et al., 1978, 1992). We suspect that these species have higher dispersal rates, which might either reflect the colonization history (i.e., the crucial dispersal events might have occurred earlier or more often than in the study species) or depend on yet unknown functional traits of these species (leading to higher SZD) (see also Sheth et al., 2020).

These findings also have important conservation implications, as they underscore the crucial importance of refugia for the long‐term survival of species with limited dispersal abilities. Refugia, in the sense of Tribsch and Schönswetter (2003), are broad geographical areas that provided suitable habitats during both cold and warm, and wet and dry stages of the Quaternary. Such areas are most likely to be found in mountainous regions with pronounced variation in topographic and microclimatic conditions. This small‐scale variation facilitates migration to suitable conditions when climate warms. Conservation efforts should thus concentrate on such areas to reduce pressure on species from other sources and thus maximize the chance of successful adaptation to climate change (Semenchuk et al., 2021). In addition, ‘assisted migration’ as a form of accelerated LDD might be considered as a last resort when all other conservation efforts fail.

## 5 CONCLUSIONS

To our best knowledge, this study is the first effort to reconstruct the post‐glacial recolonization of central Europe by plant species in a spatio‐temporally explicit manner. It provides new insights into these dynamics by highlighting the interaction between dispersal processes that operate at very different spatial scales. The five species are representatives of a considerable number of species with similarly small and often fragmented ranges, which are best explained by a combination of (i) predominant local, (ii) rare medium‐distance (up to a few kilometres) and (iii) very rare long‐distance dispersal events. As a corollary, current ranges have a strong stochastic imprint and they could just as well have a very different geographical pattern of subranges.

A successful theory of post‐glacial range formation of forest herbs should ideally provide mechanistic models that predict species distributions at various time steps and can be evaluated by scenarios derived from genetic data or fossil records if available (pollen, macrofossils). The model we use here is phenomenological, especially with respect to climate–demography relationships, and its mechanistic parts, for example, with respect to dispersal, are only roughly parameterized due to the lack of data. However, our study highlights that even an optimally parameterized and ecophysiology‐based model would not be able to predict the range dynamics of these species accurately at a spatial scale of hundreds of kilometres and temporal scale of millennia. At these scales, random events have an imprint that likely overrides the one of deterministic processes.

## AUTHOR CONTRIBUTIONS

KH, JW and WW designed the study; AG, DM and JW prepared the environmental data for the simulations, while KH compiled the species‐related information; PS and EZ performed the phylogeographical analyses; JW derived SDMs; AG adapted the modelling framework and performed the simulations; JW, WW and KH analysed model results; WW, KH, JW and SD wrote a draft version; all authors commented on the final paper.

## FUNDING INFORMATION

This work was financed by the Austrian Science Fund (FWF, project P29413 ‘Range formation of beech forest understory herbs’, project leader: Peter Schönswetter).

## CONFLICT OF INTEREST STATEMENT

The authors declare that they have no conflict of interest.

## BIOSKETCHES

**Wolfgang Willner** is a vegetation scientist interested in the current and historical biogeographical factors shaping the species composition of plant communities, with a special focus on Eurasian steppes and temperate forests.

**Johannes Wessely** is an ecologist focussing on predictions of past, current and future distributions of species under global change.

**Andreas Gattringer** is a software developer with a background in physics focussing on the development and implementation of complex modelling environments and technical applications.

## Supporting information


Data S1

Appendix S1

Video S1

## Data Availability

The data used in this study as well as the codes used for model parameterization and analysis of results are available in the Phaidra database at https://phaidra.univie.ac.at/o:1630768. Code for simulations (CATS code) is available at https://phaidra.univie.ac.at/detail/o:1635567.
