# Supplementary material for: Post‐glacial range formation of temperate forest understorey herbs – Insights from a spatio‐temporally explicit modelling approach
Source: Glob Ecol Biogeogr. 2023 Apr 10;32(7):1046–58. doi: 10.1111/geb.13677 (PMC10947399; doi:10.1111/geb.13677)
Supplement: Supplementary file 2 — Appendix S1 [file GEB-32-1046-s003.docx]

**Appendix S1. Demographic and dispersal traits of the five study species.**

Values are derived from own measurements (performed at 5–20 observation site scattered across the study area), literature and database data.

| Species name | | Mat | SP | CC | CLG | FF | JS | SY | Germ | SM | DR |
| --- | --- | --- | --- | --- | --- | --- | --- | --- | --- | --- | --- |
| *Aposeris foetida* | 0-4 | 10 / 5 | 712500 / 135000 | 1 | 0.49 / 0.38 | 0.33 / 0.17 | 14 | 0.42 / 0.42 | 1.76 | 22.32 |  |
| *Cardamine trifolia* | 1-4 | 5 / 1 | 113500 / 71500 | 1 | 0.33 / 0.10 | 0.58 / 0.41 | 45 | 0.68 / 0.48 | 1.59 | 17.95 |  |
| *Euphorbia carniolica* | 5-9 | 10 / 1 | 38850 / 2714 | 1 | 0.49 / 0.35 | 0.83 / 0.74 | 39 | 0.68 / 0.48 | 3.88 | 11.29 |  |
| *Hacquetia epipactis* | 0-4 | 1 / 1 | 230938 / 19850 | 1 | 0.63 / 0.58 | 0.33 / 0.17 | 16 | 0.68 / 0.48 | 3.07 | 5.01 |  |
| *Helleborus niger* | 1-4 | 1 / 1 | 1287500 / 500000 | 1 | 0.08 / 0.07 | 0.58 / 0.41 | 62 | 0.68 / 0.48 | 9.99 | 6.25 |  |

**Mat** – age at maturity (minimum – maximum in years); estimated as the upper/lower end of the range documented for related species (i.e. congeners or species of related genera) taken from the LEDA database (Kleyer et al. 2008) and the IntraBioDiv project (Gugerli et al. 2008).

**SP**– seed persistence in the soil seed bank (three classes assessed in years: 10 long-term persistent, 5 short-term persistent, 1 transient); estimated as the upper/lower end of the range documented for *Aposeris foetida* (Abs 1994b, a) and species related to the other five species (i.e. congeners or species of related genera) given in Thompson et al. (1993), Thompson et al. (1997), Cerabolini et al. (2007), the IntraBioDiv project (Gugerli et al. 2008) and LEDA database (Kleyer et al. 2008).

**CC**– carrying capacity (number of shoots); computed based on own measurements/estimations as number of ramets per m² * cover of species in the population (range 0-1) * coverage of populations in the cell (range 0-1) * 10^6. Estimates for the lower and upper end of the plausible range were assumed to be the 75% and 50% quantile, respectively.

**CLG**– clonal reproduction rate, i.e., number of offspring shoots per parent shoot. The CLO-PLA database (Klimešová and Bello 2009) identifies all study species (in case of *Euphorbia carniolicus* the closely related species *E. dulcis* was used) as not reproducing clonally.

**FF** – flowering frequency (% of shoots flowering per year); Values represent the 75% and 50% quantile of own measurements, respectively.

**JS** – juvenile survival (% of seedlings surviving from one year to the next); was set to 0.33/0.17 (for species reaching adult stage within 1 year), 0.58/0.41 (species maturing not before the second year) and 0.83/0.74 (species maturing not before the sixth year) (cf. Dullinger et al. 2012) representing a successful survival of every third/sixth established seedling until the reproductive (adult) stage under well-suited environmental conditions.

**SY**– seed yield (number of seeds produced per year by a flowering shoot); Values represent the 75% quantile of own measurements.

**Germ**– germination rates (percentage of germinating seeds); Species-specific data are only available for *Aposeris foetida* (Abs 1994b, a). For other species we used the 75% and 50% quantile of values for perennial, shade-tolerant non-woody plants of temperate deciduous forests with a particular focus on beech forests (Knight 1964, Ernst 1979, Nault and Gagnon 1993, Kalisz et al. 1999, Gustafsson and Ehrlén 2002, Fröborg and Eriksson 2003, Ramírez et al. 2006, Mondoni et al. 2009, Christensen and Gorchov 2010, Kolb and Barsch 2010, Kolb et al. 2010, Oborny et al. 2011, Garrido et al. 2012, Kosiński 2012, Ehrlén et al. 2015).

**SM** – seed mass (mg) was determined by own measurements supplemented by data taken from the LEDA database (Kleyer et al. 2008) and the BiolFlor database (Klotz et al. 2002).

**DR** – hourly detachment rate (%) of seeds from fur of a “general large mammalian seed dispersal vector” representing a mixture of detachment rates from sheep/bear (20%) and cattle/deer/rabbit/etc. fur (80%), respectively, as calculated from the seed mass and surface structure by the regression equations in Römermann et al. (2005). Seed surface structure was classified from own samples (minimum *n*=10) of seeds for all species.

**References:**

Abs, C. (1994a). Populationsökologie als geobotanische Kausalanalyse. Berichte der Reinhold-Tüxen-Gesellschaft, 6, 149–163.

Abs, C. (1994b). Populationsökologie von *Aposeris foetida* (L.) Less: Standortbedingte Modifikationen des Lebenszyklus und das Wirkgefüge bei der Etablierung in verschiedenen Pflanzengesellschaften. Dissertation, Geobotanica-Verlag, Fürhlzen.

Cerabolini, B., Ceriani, R. M., Caccianiga, M., Andreis, R. D., & Raimondi, B. (2007). Seed size, shape and persistence in soil: a test on Italian flora from Alps to Mediterranean coasts. Seed Science Research, 13, 75–85.

Christensen, D. L., & Gorchov, D. L. (2010). Population dynamics of goldenseal (*Hydrastis canadensis*) in the core of its historical range. Plant Ecology, 210, 195–211.

Dullinger, S., Gattringer, A. , Thuiller, W., Moser, D. , Zimmermann, N. E., Guisan, A., Willner, W., Plutzar, C., Leitner, M., Mang, T., Caccianiga, M., Dirnböck, T., Ertl, S., Fischer, A., Lenoir, J., Svenning, J.-C., Psomas, A., Schmatz, D. R., Silc, U., Vittoz, P., & Hülber, K. (2012). Extinction debt of high-mountain plants under twenty-first-century climate change. Nature Climate Change, 2, 619–622.

Ehrlén, J., Raabova, J., & Dahlgren, J. P. (2015). Flowering schedule in a perennial plant; life-history trade-offs, seed predation, and total offspring fitness. Ecology, 96, 2280–2288.

Ernst, W. H. O. (1979). Population biology of *Allium ursinum* in northern Germany. Journal of Ecology, 67, 347–362.

Fischer, M. A., Oswald, K., & Adler, W. (2008). Exkursionsflora für Österreich, Liechtenstein und Südtirol. Land Oberösterreich, Linz.

Fröborg, H., & Eriksson, O. (2003). Predispersal seed predation and population dynamics in the perennial understorey herb *Actaea spicata*. Canadian Journal of Botany, 81, 1058–1069.

Garrido, J. L., Rey, P. J., Herrera, C. a. M., & Ramírez, J. M. (2012). Negative evidence of local adaptation to the establishment conditions in a perennial herb. Plant Ecology, 213, 1555–1569.

Gugerli, F., Englisch, T., Niklfeld, H., Tribsch, A., Mirek, Z., Ronikier, M., Zimmermann, N. E., Holderegger, R., Taberlet, P. & C. IntraBioDiv. (2008). Relationships among levels of biodiversity and the relevance of intraspecific diversity in conservation - a project synopsis. Perspectives in Plant Ecology Evolution and Systematics, 10, 259–281.

Gustafsson, C., & Ehrlén, J. (2002). Seed availability and recruitment of the perennial herb *Sanicula europaea*. Ecoscience, 9, 526–532.

Kalisz, S., Hanzawa, F. M., Tonsor, S. J., Thiede, D. A., & Voigt, S. (1999). Ant-mediated seed dispersal alters pattern of relatedness in a population of *Trillium grandiflorum*. Ecology, 80, 2620–2634.

Katul, G. G., Porporato, A., Nathan, R., Siqueira, M., Soons, M. B., Poggi, D., Horn, H. S. & Levin, S. A. (2005). Mechanistic analytical models for long-distance seed dispersal by wind. American Naturalist, 166, 368–381.

Kleyer, M., Bekker, R. M., Knevel, I. C., Bakker, J. P., Thompson, K., Sonnenschein, M., Poschlod, P., van Groenendael, J. M., Klimeš, L., Klimešová, J., Klotz, S., Rusch, G. M., Hermy, M., Adriaens, D., Boedeltje, G., Bossuyt, B., Dannemann, A., Endels, P., Götzenberger, L., Hodgson, J. G., Jackel, A. K., Kühn, I., Kunzmann, D., Ozinga, W. A., Römermann, C., Stadler, M., Schlegelmilch, J., Steendam, H. J., Tackenberg, O., Wilmann, B., Cornelissen, J. H. C., Eriksson, O., Garnier, E., & Peco, B. (2008). The LEDA Traitbase: a database of life-history traits of the Northwest European flora. Journal of Ecology, 96, 1266–1274.

Klimešová, J., & Bello, F. (2009). CLO-PLA: The database of clonal and bud bank traits of Central European flora. Journal of Vegetation Science, 20, 511–516.

Klotz, S., Kühn, I., & Durka, W. (2002). BIOLFLOR - Eine Datenbank mit biologisch-ökologischen Merkmalen zur Flora von Deutschland. Landwirtschaftsverlag, Münster.

Knight, G. H. (1964). Some factors affecting the distribution of *Endymion nonscriptus*. Journal of Ecology, 52, 405–421.

Kolb, A., & Barsch, K. (2010). Environmental factors and seed abundance influence seedling emergence of a perennial forest herb. Acta Oecologica, 36, 507–513.

Kolb, A., Dahlgren, J. P., & Ehrlen, J. (2010). Population size affects vital rates but not population growth rate of a perennial plant. Ecology, 91, 3210–3217.

Kosiński, I. (2012). Generative reproduction dynamics in populations of the perennial herb *Polygonatum multiflorum* (Asparagaceae). Annales Botanici Fennici, 49, 217–228.

Mondoni, A., Probert, R., Rossi, G., & Hay, F. (2009). Habitat-related germination behaviour and emergence phenology in the woodland geophyte *Anemone ranunculoides* L. (Ranunculaceae) from northern Italy. Seed Science Research, 19, 137–144.

Nault, A., & Gagnon, D. (1993). Ramet demography of *Allium tricoccum*, a spring ephemeral, perennial forest herb. Journal of Ecology, 81, 101–119.

Oborny, B., Botta-Dukát, Z., Rudolf, K., & Morschhauser, T. (2011). Population ecology of *Allium ursinum*, a space-monopolizing clonal plant. Acta Botanica Hungarica, 53, 371–388.

Ramírez, J. M., Rey, P. J., Alcántara, J. M., & Sánchez-Lafuente, A. M. (2006). Altitude and woody cover control recruitment of *Helleborus foetidus* in a Mediterranean mountain area. Ecography, 29, 375–384.

Römermann, C., Tackenberg, O., & Poschlod, P. (2005). How to predict attachment potential of seeds to sheep and cattle coat from simple morphological seed traits. Oikos, 110, 219–230.

Skarpaas, O., & Shea, K. (2007). Dispersal Patterns, Dispersal Mechanisms, and Invasion Wave Speeds for Invasive Thistles. The American Naturalist, 170, 421–430.

Thompson, K., Bakker, J. P., & Bekker, R. M. (1997(. The Soil Seed Banks of North West Europe: Methodology, Density and Longevity. Cambridge University Press, Cambridge.

Thompson, K., Band, S. R., & Hodgson, J. G. (1993). Seed size and shape predict persistence in soil. Functional Ecology, 7, 236-241.

**Appendix S2. Sampling sites of the five study species.**

Population ID; country; region; locality; altitude (m a.s.l.); coordinates; collector; date.

***Aposeris foetida***

Af1; Slovenia; Gorenjska; Karavanke mountains, between the mountain hut Valvasorjev dom and the pasture Žirovniška planina; 1269; 14°9'25''E, 46°25'29''N; Spela Novak; 03.8.2016

Af2; Slovenia; Gorenjska; Kamniško-Savinjske Alpe, 0.01 km S of the big parking place in Ravenska Kočna; 1174; 14°32'9''E, 46°22'59''N; Spela Novak; 07.8.2016

Af3; Slovenia; Gorenjska; Kamniško-Savinjske Alpe, Potoče, 0.5 km NW of Javorov vrh; 1320; 14°27'37''E, 46°19'38''N; Spela Novak; 15.9.2016

Af6; Croatia; Krapina-Zagorje; Medvenica, Lojstekov put, in the vicinity of Adolfovac; ; 15°58'54''E, 45°53'14''N; Ivana Rešetnik; 17.9.2016

Af5; Slovenia; Gorenjska; Kamniško-Savinjske Alpe, slope of Kriška gora, Zgornje Vetrno, 0.050 km E of the parking place for "strma pot na Kriško goro"; 850; 14°19'6''E, 46°20'39''N; Spela Novak; 30.10.2016

Af7; Italy; Friuli-Venezia Giulia; Alpi Giulie, immediately N of the village Riofreddo; 860; 13°34'5''E, 46°28'17''N; Christoph Dobeš; 09.6.2017

Af8; Italy; Friuli-Venezia Giulia; SW side of mount Cullata, 1.8 km E of Dierico; 830; 13°9'11''E, 46°30'51''N; Christoph Dobeš; 10.6.2017

Af9; Italy; Friuli-Venezia Giulia; Val Venzonassa, immediately N of Forca Tacia W of Musi; 1080; 13°14'23''E, 46°18'48''N; Christoph Dobeš; 11.6.2017

Af10; Italy; Veneto; 1.0 km SE of Cima Sappada, E of the serpentine of the main road; 1160; 12°44'3''E, 46°34'1''N; Christoph Dobeš; 11.6.2017

Af11; Italy; Friuli-Venezia Giulia; 1.3 km SW of the village Shgittosa, along the street to the Forchia die Meduno; 560; 12°48'16''E, 46°15'3''N; Christoph Dobeš; 12.6.2017

Af12; Italy; Veneto; 0.4 km SW of Fener (W Valdobbiadene); 350; 11°56'22''E, 45°54'8''N; Christoph Dobeš; 13.6.2017

Af13; Italy; Veneto; 0.7 km S of Voltago; 850; 12°0'22''E, 46°16'0''N; Christoph Dobeš; 14.6.2017

Af14; Italy; Trentino; Val di Sella, localitá Dosso 3 km ENE Sella and 4 km S of Borgo Valsugana; 770; 11°26'56''E, 46°1'4''N; Christoph Dobeš; 15.6.2017

Af15; Italy; Veneto; Altopiano dei Setti Communi, 2.0 km W of San Antonio, S of the main road; 1050; 11°11'17''E, 45°45'20''N; Christoph Dobeš; 16.6.2017

Af16; Italy; Trentino; 0.6 km SW of Tregiovo; 960; 11°2'49''E, 46°26'8''N; Christoph Dobeš; 16.6.2017

Af17; Italy; Trentino; Brenta mountains, 1.2 km NE of Binio; 1200; 10°46'12''E, 46°4'26''N; Christoph Dobeš; 17.6.2017

Af18; Italy; Lombardia; Adamello Mountains, Valle del Caffaro, 2.9 km NNE of Valle Dorizzo; 1410; 10°27'48''E, 45°54'25''N; Christoph Dobeš; 18.6.2017

Af19; Italy; Lombardia; Spiazzi di Gromo, immediately E of the parking place (E of Boario Spiazzi); 1210; 9°57'51''E, 45°58'18''N; Christoph Dobeš; 19.6.2017

Af20; Italy; Lombardia; Val di Faggio, 0.5 km SE of Moggio; 1040; 9°30'4''E, 45°55'23''N; Christoph Dobeš; 20.6.2017

Af21; Switzerland; Ticino; 2.1 km E of Cadro, Alpe Bolla; 1170; 9°0'39''E, 46°2'20''N; Christoph Dobeš; 21.6.2017

Af22; Switzerland; Valais; Rhone valley, 0.4 km NW of Mollens; 1150; 7°31'6''E, 46°19'7''N; Christoph Dobeš; 07.7.2017

Af25; Switzerland; Bern; 2.5 km SE of Gstaad; 1500; 7°18'4''E, 46°27'9''N; Christoph Dobeš; 08.7.2017

Af24; Switzerland; Fribourg; Niremont, 0.4 km NW of the hamlet Rathwel; 1330; 6°58'36''E, 46°32'51''N; Christoph Dobeš; 08.7.2017

Af23; Switzerland; Valais; Vallon de Nant, 1.4 km SE of Plans-sur-Bex; 1280; 7°6'29''E, 46°14'56''N; Christoph Dobeš; 08.7.2017

Af26; Switzerland; Bern; Simmen valley, 1.5 km SW of Erlenbach; 800; 7°32'9''E, 46°39'14''N; Christoph Dobeš; 10.7.2017

Af116; Ukraine; Chernivtsi Oblast; Eastern Carpathians, Cheremoskyi National Nature Park: Chornyi Dil mountain range, c. 3.6 km NW of Sarata; 1447; 24°57'42''E, 47°46'14''N; Clemens Pachschwöll & Tetiana Pochynok; 25.7.2017

Af27; Italy; Tuscany; 2.9 km SW from Vinca; 1221; 10°7'25''E, 44°7'35''N; Ernesto Venturi; 13.5.2017

Af72; Romania; Hunedoara; 1.7 km WbN from Boholt; 278; 22°56'40''E, 45°56'46''N; Mislav Vulelija; 13.7.2017

Af28; Italy; Tuscany; 2.3 km NW from Levigliani; 1203; 10°16'51''E, 44°2'32''N; Ernesto Venturi; 13.5.2017

Af29; Italy; Tuscany; 1.2 km W from Arni; 1065; 10°14'0''E, 44°3'57''N; Ernesto Venturi; 31.5.2017

Af108; Slovenia; Nova Gorica; 1 km W of Vogrsko on Highway H4, go 0.5 km N; 100; 13°41'6''E, 45°55'1''N; Simon Stifter; 23.5.2017

Af82; Poland; Subcarpathia; Gora Sobien, 3 km E of Zaluz; 370; 22°19'40''E, 49°31'36''N; Simon Stifter; 05.6.2017

Af109; Ukraine; Khersonska oblast; Krushelnytsia, on the N exposed hillfoot ; 443; 23°29'11''E, 49°6'10''N; Simon Stifter; 09.6.2017

Af100; Romania; Arad; 8.5 km into the Valley E of Dezna; 630; 22°21'11''E, 46°25'38''N; Simon Stifter; 11.8.2017

Af124; Italy; Friuli-Venezia Giulia; Val d'Arzino, along the street close to Fraponti; 335; 12°57'49''E, 46°16'16''N; Simon Stifter; 31.8.2019

Af101; Slovenia; Tolmin; 1 km S of Sedlo, close to Bela river; 373; 13°26'32''E, 46°14'43''N; Simon Stifter; 24.5.2017

Af78; Poland; Subcarpathia; behind the war memorial, Baligrod; 500; 22°16'42''E, 49°19'14''N; Simon Stifter; 06.6.2017

Af110; Ukraine; Lvivska oblast; 2 Km S of Pshonets; 709; 23°23'11''E, 48°52'60''N; Simon Stifter; 09.6.2017

Af83; Romania; Caraș-Severin; 2 km NE of Rachita (Sebes); 400; 23°31'59''E, 45°53'36''N; Simon Stifter; 12.8.2017

Af102; Slovenia; Tolmin; Klavze, 0.25 km S from baca river; 273; 13°48'50''E, 46°9'30''N; Simon Stifter; 26.5.2017

Af79; Poland; Subcarpathia; Muczne, on the way to Mount Kudriawynski Wierch; 950; 22°46'32''E, 49°6'42''N; Simon Stifter; 06.6.2017

Af111; Ukraine; Zakarpatska oblast; hill NE of Kolchyno; 200; 22°45'55''E, 48°28'38''N; Simon Stifter; 09.6.2017

Af84; Romania; Sibiu; 0.5 km N of Cornatel (Sibiu) take the forest road on the left and follow for 2 km; 500; 24°20'25''E, 45°49'26''N; Simon Stifter; 13.8.2017

Af103; Slovenia; Škofja Loka; E of Praprotno get over the river, take road on the left for 0.3 km, forest on E; 446; 14°15'55''E, 46°11'50''N; Simon Stifter; 26.5.2017

Af80; Poland; Subcarpathia; SE of Roztoki Gorne, along the border/path; 850; 22°19'56''E, 49°8'43''N; Simon Stifter; 06.6.2017

Af112; Ukraine; Zakarpatska oblast; E exposed hillfoot, Dibrova Reserve, Belykyi; 377; 24°1'26''E, 48°0'3''N; Simon Stifter; 10.6.2017

Af85; Romania; Alba; N of Valea Lunga (Blaj), take road to NW vor 1 km; 380; 24°2'32''E, 46°8'44''N; Simon Stifter; 13.8.2017

Af104; Slovenia; Cerknica; on Mt. Slivnica (Cerknica) behind the Hut: Dom na Slivnici; 1093; 14°24'22''E, 45°47'22''N; Simon Stifter; 27.5.2017

Af81; Poland; Subcarpathia; 4 Km E of Tylawa, 0.2 km E from the campsite; 430; 21°43'57''E, 49°27'42''N; Simon Stifter; 07.6.2017

Af113; Ukraine; Ivano-Frankivska oblast; 4 km E of Mykulychyn; 900; 24°38'27''E, 48°25'10''N; Simon Stifter; 11.6.2017

Af86; Romania; Bistrița-Năsăud; NE of Valea Mare (CV); 730; 26°1'43''E, 45°46'15''N; Simon Stifter; 15.8.2017

Af114; Ukraine; Chernivetska oblast; forest S of Sniachiv; 330; 25°50'27''E, 48°10'35''N; Simon Stifter; 13.6.2017

Af105; Slovenia; Žalec; close to Jama Pekel cave ; 350; 15°7'58''E, 46°17'24''N; Simon Stifter; 28.6.2017

Af87; Romania; Alba; 1 km N of Garbova de Jos; 380; 23°40'11''E, 46°16'11''N; Simon Stifter; 17.8.2017

Af115; Ukraine; Ivano-Frankivska oblast; forest S of Halych, N of Kozyna; 300; 24°43'37''E, 49°5'42''N; Simon Stifter; 14.6.2017

Af106; Slovenia; Črnomelj; from Crmosnjece go to Komarna Vas Farm, 1 km NW; 630; 15°4'5''E, 45°40'55''N; Simon Stifter; 29.6.2017

Af88; Romania; Cluj; small forest between Rediu and Gheorghieni (Cluj-Nopoca); 680; 23°42'37''E, 46°41'29''N; Simon Stifter; 17.8.2017

Af107; Slovenia; Kočevje; 1.5 km S of Borovec pri Kocevski Reki; 800; 14°46'52''E, 45°32'12''N; Simon Stifter; 30.6.2017

Af89; Romania; Argeș; 1.5 km NNW of Badesti; 650; 23°37'28''E, 46°59'19''N; Simon Stifter; 17.8.2017

Af90; Romania; Alba; W of Baia de Aries, 0.5 km N of the street; 560; 23°16'8''E, 46°22'56''N; Simon Stifter; 17.8.2017

Af91; Romania; Bihor; 1 km SE of Bucuroaia; 300; 22°11'36''E, 46°54'59''N; Simon Stifter; 18.8.2017

Af92; Romania; Sălaj; N of Nusfalau pass the railway and folow the street for 1 km until the Hotel; 280; 22°44'23''E, 47°12'37''N; Simon Stifter; 19.8.2017

Af93; Romania; Olt; 2 km SE of Chilia, at the foot of Culmea Codrului Mountains; 240; 23°6'8''E, 47°37'33''N; Simon Stifter; 19.8.2017

Af94; Romania; Satu Mare; from Turt follow the Street 3km and take road on the right for 1 km to reach the forest; 350; 23°11'7''E, 48°1'5''N; Simon Stifter; 20.8.2017

Af95; Romania; Maramureș; Baia Mare, Valea rosie; 470; 23°33'35''E, 47°40'31''N; Simon Stifter; 20.8.2017

Af96; Romania; Maramureș; Costitui: forest behind castelul Apafi; 470; 24°1'31''E, 47°52'36''N; Simon Stifter; 20.8.2017

Af97; Romania; Bistrița-Năsăud; S of Nasaud, after the bridge take the small road on the right and follow for 2 km; 380; 24°23'58''E, 47°16'17''N; Simon Stifter; 21.8.2017

Af98; Romania; Cluj; from Rugasesti take the street to Salatruc, after 0.5 km on left hand side; 350; 23°51'55''E, 47°14'50''N; Simon Stifter; 21.8.2017

Af99; Romania; Suceava; Reservatie Stiintifica; 380; 26°11'47''E, 47°44'55''N; Simon Stifter; 23.8.2017

Af45; Slovakia; Prešov; Šarišská vrchovina-Stráže Mts, near the village Kanaš; 332; 21°12'58''E, 49°3'16''N; J. Kochjarová, R. Hrivnák, M. Slezák; 06.7.2017

Af46; Slovakia; Prešov; Slanské vrchy Mts, near the village Lipníky; 330; 21°25'9''E, 49°3'42''N; J. Kochjarová, R. Hrivnák, M. Slezák; 06.7.2017

Af47; Slovakia; Prešov; Ondavská vrchovina Mts, near the village Radoma; 251; 21°34'45''E, 49°11'35''N; J. Kochjarová, R. Hrivnák, M. Slezák; 06.7.2017

Af48; Slovakia; Prešov; Laborecká vrchovina Mts, near the village Vislava; 303; 21°40'24''E, 49°16'23''N; J. Kochjarová, R. Hrivnák, M. Slezák; 06.7.2017

Af49; Slovakia; Prešov; Laborecká vrchovina Mts, near the village Pstriná; 319; 21°45'36''E, 49°17'38''N; J. Kochjarová, R. Hrivnák, M. Slezák; 06.7.2017

Af73; Serbia; Pećki okrug; Bajina Bašta, Rača Monastery; 366; 19°32'30''E, 43°55'51''N; Nevena Kuzmanović; 12.5.2017

Af117; Bosnia and Herzegovina; Federation of Bosnia and Herzegovina; Glavatičevo; 670; 18°5'43''E, 43°29'41''N; Bogunić Faruk; 19.5.2017

Af74; Serbia; Mačvanski okrug; Banja Koviljača, Gučevo; 553; 19°9'44''E, 44°29'31''N; Nevena Kuzmanović; 13.5.2017

Af118; Bosnia and Herzegovina; Federation of Bosnia and Herzegovina; Brateljevići; 770; 18°38'39''E, 44°11'17''N; Bogunić Faruk; 23.6.2017

Af75; Serbia; Mačvanski okrug; Gornje Košlje, gorge of river Trešnjica; 349; 19°32'39''E, 44°8'39''N; Nevena Kuzmanović, Ivana Janković, Tijana Milekić; 19.5.2017

Af119; Bosnia and Herzegovina; Federation of Bosnia and Herzegovina; Puzim; 1575; 18°15'52''E, 43°35'21''N; Bogunić Faruk; 23.5.2017

Af120; Bosnia and Herzegovina; Federation of Bosnia and Herzegovina; Suha; 670; 18°39'42''E, 43°18'54''N; Bogunić Faruk; 09.4.2017

Af76; Serbia; Zlatiborski okrug; Tara, Predov krst; 1030; 19°17'54''E, 43°56'19''N; Nevena Kuzmanović, Tijana Milekić; 22.8.2017

Af121; Bosnia and Herzegovina; Federation of Bosnia and Herzegovina; Duboštica; 680; 18°18'58''E, 44°14'29''N; Bogunić Faruk; 01.6.2017

Af77; Bosnia and Herzegovina; Republika Srpska; Smolin, Veliki Smolin; 1000; 17°52'60''E, 44°27'54''N; Gordana Tomović, Ksenija Jakovljević, Uroš Buzurović; 08.7.2017

Af122; Bosnia and Herzegovina; Federation of Bosnia and Herzegovina; Džepska planina; 1020; 18°2'56''E, 43°39'56''N; Bogunić Faruk; 04.5.2017

Af123; Bosnia and Herzegovina; Republika Srpska; Liplje; 640; 17°37'14''E, 44°36'12''N; Bogunić Faruk; 26.9.2017

Af50; Slovenia; Tržič; Karawank nahe Crna; 798; 14°47'14''E, 46°28'14''N; Julian Haider; 28.5.2017

Af59; Germany; Bavaria; gorge near Sankt Valentin; 700; 12°39'36''E, 47°43'13''N; Julian Haider; 30.6.2017

Af60; Austria; Tyrol; Kufstein, next to Kaiserbach; 559; 12°11'13''E, 47°35'19''N; Julian Haider; 01.7.2017

Af61; Austria; Tyrol; Stans; 869; 11°41'54''E, 47°22'32''N; Julian Haider; 01.7.2017

Af62; Germany; Bavaria; Miesbach; 687; 11°52'48''E, 47°46'39''N; Julian Haider; 02.7.2017

Af63; Germany; Bavaria; Wolfshausen an der Isar; 570; 11°26'48''E, 47°55'25''N; Julian Haider; 02.7.2017

Af64; Austria; Tyrol; Gießenbach; 1025; 11°13'52''E, 47°22'1''N; Julian Haider; 03.7.2017

Af65; Germany; Bavaria; Halbech; 838; 10°49'15''E, 47°37'25''N; Julian Haider; 04.7.2017

Af66; Germany; Bavaria; near Dietersberg; 869; 10°16'37''E, 47°22'33''N; Julian Haider; 02.7.2017

Af67; France; Haute-Savoie; Lac de Vallon; 1084; 6°34'2''E, 46°12'45''N; Julian Haider; 07.7.2017

Af68; France; Savoie; gorge near Ecole; 847; 6°11'28''E, 45°38'24''N; Julian Haider; 07.7.2017

Af51; Slovenia; Tržič; Trzic; 806; 14°20'20''E, 46°23'52''N; Julian Haider; 29.5.2017

Af69; France; Isere; near Grenoble; 1089; 5°38'2''E, 45°5'49''N; Julian Haider; 08.7.2017

Af70; France; Drôme; Vassieux; 1368; 5°17'41''E, 44°54'41''N; Julian Haider; 08.7.2017

Af71; France; Hautes-Alpes; Ancelle; 1384; 6°13'27''E, 44°37'12''N; Julian Haider; 11.7.2017

Af52; Slovenia; Jesenice; Kranjska Gora; 903; 13°45'6''E, 46°29'14''N; Julian Haider; 30.5.2017

Af53; Austria; Carinthia; Stockenboi, near the water fall; 899; 13°27'35''E, 46°42'13''N; Julian Haider; 31.5.2017

Af54; Austria; Salzburg; Werfen near the mine; 560; 13°9'52''E, 47°30'34''N; Julian Haider; 01.6.2017

Af55; Austria; Salzburg; Enzing; 576; 13°11'46''E, 47°54'60''N; Julian Haider; 01.6.2017

Af56; Austria; Upper Austria; Bad Ischl; 976; 13°36'38''E, 47°41'53''N; Julian Haider; 01.6.2017

Af57; Austria; Upper Austria; Wankham; 419; 13°44'24''E, 47°59'32''N; Julian Haider; 01.6.2017

Af58; Austria; Upper Austria; along the river Traun S of the city of Traun; 273; 14°15'26''E, 48°13'8''N; Julian Haider; 29.6.2017

Af31; Croatia; Karlovac; Generalski Stol; ; 15°22'48''E, 45°21'12''N; D. Škrtić; 18.6.2017

Af32; Croatia; Koprivnica-Krizevci; Kalnik; ; 16°27'29''E, 46°68'0''N; Ivana Rešetnik; 14.7.2017

Af40; Croatia; Pozega-Slavonia; Krndija, near Petrov vrh; ; 17°52'21''E, 45°28'53''N; I. Rešetnik, M. Doboš; 29.4.2017

Af41; Croatia; Primorje-Gorski Kotar; Mala Lešnica; ; 14°51'9''E, 45°26'17''N; Ivana Rešetnik; 23.6.2017

Af37; Croatia; Krapina-Zagorje; Medvednica, Glavica; ; 15°53'9''E, 45°51'2''N; Ivana Rešetnik; 09.4.2017

Af39; Croatia; Bjelovar-Bilogora; Moslavačka gora, Garič grad; ; 16°45'23''E, 45°37'49''N; Ivana Rešetnik; 30.4.2017

Af34; Croatia; Primorje-Gorski Kotar; NP Risnjak, Medvjeđa vrata; ; 14°37'22''E, 45°24'53''N; Ivana Rešetnik; 24.6.2017

Af42; Croatia; Karlovac; Ogulin, Otok Oštarijski, Veljun; ; 15°14'41''E, 45°13'15''N; Ivana Rešetnik; 23.6.2017

Af44; Croatia; Lika-Senj; Plitvice, Gornji Babin Potok-Končarev kraj; ; 15°33'18''E, 44°49'39''N; Ivana Rešetnik; 22.6.2017

Af38; Croatia; Sisak-Moslavina; Prkovec; ; 16°5'43''E, 45°34'16''N; Ivana Rešetnik; 01.4.2017

Af35; Croatia; Zagreb; Samoborsko gorje, Žužići; ; 15°39'1''E, 45°46'43''N; Ivana Rešetnik; 14.4.2017

Af43; Croatia; Krapina-Zagorje; Strahinjčica, Radoboj; ; 15°55'39''E, 46°10'55''N; Ivana Rešetnik; 13.5.2017

Af30; Croatia; Primorje-Gorski Kotar; Učka, Vojak; ; 14°12'27''E, 45°17'59''N; I. Rešetnik, S. Bogdanović, M. Temunović; 23.7.2017

Af33; Croatia; Lika-Senj; Velebit, Premužićeva staza; ; 14°59'6''E, 44°47'40''N; I. Rešetnik, P. Schönswetter; 27.6.2017

Af36; Croatia; Zagreb; Žumberak, Sopotski slap; ; 15°23'49''E, 45°45'52''N; Ivana Rešetnik; 14.4.2017

***Cardamine trifolia***

Ct1; Slovenia; Gorenjska; Kamniško-Savinjske Alpe, 0.01 km S of the big parking place in Ravenska Kočna; 1174; 14°32'9''E, 46°22'59''N; Spela Novak; 07.8.2016

Ct2; Slovenia; Gorenjska; Julian Alps, Stara Fužina, 0.3 km W of the parking place on planina Blato; 1200; 13°50'43''E, 46°18'41''N; Spela Novak; 09.8.2016

Ct3; Austria; Lower Austria; Northern Calcareous Alps, Dürrenstein, Seetal, E of the Mittersee, left and right of the service road; 770; 15°4'35''E, 47°49'40''N; Christoph Dobeš; 27.8.2016

Ct5; Croatia; Primorje-Gorski Kotar; Gorski Kotar, Samarske stijene, in the vicinity of Ratkovo skloniste; ; 14°57'8''E, 45°13'33''N; Ivana Rešetnik; 03.9.2016

Ct4; Slovenia; Gorenjska; Kamniško-Savinjske Alpe, Logarska dolina, 0.1 km E of the hut Koča na Klemenči jami; 1220; 14°38'7''E, 46°22'43''N; Spela Novak; 04.11.2016

Ct6; Italy; Friuli-Venezia Giulia; Alpi Giulie, immediately N of the village Riofreddo; 860; 13°34'5''E, 46°28'17''N; Christoph Dobeš; 09.6.2017

Ct7; Italy; Friuli-Venezia Giulia; north side of mount Tersadia, 2.8 km W of Paularo and 1 km S of Ligosullo; 1130; 13°4'45''E, 46°31'50''N; Christoph Dobeš; 10.6.2017

Ct9; Italy; Veneto; 1.0 km SE of Cima Sappada, E of the serpentine of the main road; 1160; 12°44'3''E, 46°34'1''N; Christoph Dobeš; 11.6.2017

Ct8; Italy; Friuli-Venezia Giulia; Val Venzonassa, 0.9 km E of Forca Tacia and 1.7 km W of Musi; 900; 13°15'2''E, 46°18'56''N; Christoph Dobeš; 11.6.2017

Ct10; Italy; Friuli-Venezia Giulia; 0.7 km SW of the village Shgittosa, along the street to the Forchia die Meduno; 460; 12°48'43''E, 46°15'12''N; Christoph Dobeš; 12.6.2017

Ct11; Italy; Veneto; Cima Alta, 2.7 km WNW Susegana and 0.7 km NE of San Daniele-tombola; 100; 12°13'6''E, 45°51'25''N; Christoph Dobeš; 12.6.2017

Ct12; Italy; Veneto; 6.2 km S of Seren del Grappa, Misola; 730; 11°50'3''E, 45°56'4''N; Christoph Dobeš; 13.6.2017

Ct13; Italy; Veneto; Altopiano dei Setti Communi, 2.1 km SW of Roana; 910; 11°26'60''E, 45°51'39''N; Christoph Dobeš; 15.6.2017

Ct14; Italy; Veneto; Altopiano dei Setti Communi, 2.0 km W of San Antonio, S of the main road; 1050; 11°11'17''E, 45°45'20''N; Christoph Dobeš; 16.6.2017

Ct15; Switzerland; Bern; 2.5 km SE of Gstaad; 1500; 7°18'4''E, 46°27'9''N; Christoph Dobeš; 08.7.2017

Ct89; Austria; Styria; Eselsbergbach NW of Oberwölz; 1170; 14°11'29''E, 47°14'0''N; Th. Zimmermann; 02.7.2019

Ct91; Austria; Styria; 2.5 km E of Krakaudorf, S of the main road; 1100; 14°3'15''E, 47°10'56''N; Th. Zimmermann; 15.7.2019

Ct92; Austria; Styria; E of the southbound main road descending from the Sölkpass, E of the Dorferhütte; 1280; 14°5'3''E, 47°14'33''N; Th. Zimmermann; 15.7.2019

Ct90; Austria; Styria; 3.4 km ENE of Stadl an der Mur, SE of the Falkenhütte; 1390; 14°1'42''E, 47°5'29''N; Th. Zimmermann; 15.7.2019

Ct16; Italy; Tuscany; 0.7 km S Passo die Lupatti; 1126; 11°54'9''E, 43°48'7''N; Ernesto Venturi; 23.4.2017

Ct17; Italy; Emilia-Romagna; 1.4 km NW from Campigna; 1182; 11°44'10''E, 43°52'28''N; Ernesto Venturi; 07.5.2017

Ct18; Italy; Emilia-Romagna; 1 km E from Campigna; 1279; 11°44'5''E, 43°52'10''N; Ernesto Venturi; 07.5.2017

Ct19; Italy; Emilia-Romagna; 0.7 km SE from Campigna; 1187; 11°44'24''E, 43°52'7''N; Ernesto Venturi; 08.5.2017

Ct20; Italy; Tuscany; 3.3 km E from Il Castagno D'Andrea; 1358; 11°42'6''E, 43°53'25''N; Ernesto Venturi; 08.5.2017

Ct21; Italy; Emilia-Romagna; Rigoso, 0.2 km NW from Lago Squincio; 1257; 10°8'6''E, 44°21'19''N; Ernesto Venturi; 07.6.2017

Ct22; Italy; Emilia-Romagna; Rigoso, 0.6 km W from Lago Palo; 1453; 10°6'20''E, 44°21'27''N; Ernesto Venturi; 07.6.2017

Ct23; Italy; Emilia-Romagna; Rigoso, 0.1 km NW from Lago Ballano; 1368; 10°5'56''E, 44°22'11''N; Ernesto Venturi; 08.6.2017

Ct78; Czech Republic; Vysočina; Chlébské; ; 16°23'21''E, 49°28'34''N; Bohumil Mandák;

Ct79; Czech Republic; Moravia-Silesia; Lišková; ; 18°26'53''E, 49°24'10''N; Bohumil Mandák;

Ct80; Czech Republic; Vysočina; Nové Ransko; ; 15°47'39''E, 49°40'17''N; Bohumil Mandák;

Ct81; Czech Republic; South Bohemia; Chlum o Třeboně; ; 14°56'44''E, 48°55'24''N; Bohumil Mandák;

Ct82; Czech Republic; South Bohemia; Hojná Voda; ; 14°45'12''E, 48°42'21''N; Bohumil Mandák;

Ct76; Slovenia; Nova Gorica; 1 km W of Vogrsko on Highway H4, go 0.5 km N; 100; 13°41'6''E, 45°55'1''N; Simon Stifter; 23.5.2017

Ct68; Poland; Silesia; Pewel mala, in the forest behind the Kaplica Kielbasow Monument; 420; 19°17'35''E, 49°39'11''N; Simon Stifter; 03.6.2017

Ct93; Italy; Friuli-Venezia Giulia; Val d'Arzino, along the street close to Fraponti; 335; 12°57'49''E, 46°16'16''N; Simon Stifter; 31.8.2019

Ct77; Slovenia; Tolmin; 1 km S of Sedlo, close to Bela river; 373; 13°26'32''E, 46°14'43''N; Simon Stifter; 24.5.2017

Ct69; Poland; Silesia; 1.5 km SSE of Soblowka beside the River; 670; 19°8'59''E, 49°25'44''N; Simon Stifter; 03.6.2017

Ct70; Slovenia; Tolmin; Klavze, 0.25 km S from baca river; 275; 13°49'4''E, 46°9'23''N; Simon Stifter; 26.5.2017

Ct66; Poland; Lesser Poland; 3 km SW of Siwa Polana parking place; 960; 19°49'13''E, 49°16'18''N; Simon Stifter; 04.6.2017

Ct71; Slovenia; Škofja Loka; E of Praprotno get over the river, take road on the left for 0.3 km, forest on E; 570; 14°16'13''E, 46°11'47''N; Simon Stifter; 26.5.2017

Ct67; Poland; Lesser Poland; Polana Mlyniska, Zakopane; 920; 19°56'24''E, 49°16'43''N; Simon Stifter; 04.6.2017

Ct72; Slovenia; Ajdovščina; between Podkraj and Kalce in rocky forest; 839; 14°8'16''E, 45°52'0''N; Simon Stifter; 27.5.2017

Ct73; Slovenia; Ptuj; 2 km S of Kozminci; 300; 15°51'17''E, 46°16'56''N; Simon Stifter; 27.6.2017

Ct74; Slovenia; Črnomelj; from Crmosnjece go to Resa, 3 km S, close to the forest road; 950; 15°2'45''E, 45°38'18''N; Simon Stifter; 29.6.2017

Ct75; Slovenia; Kočevje; 7 km W of Borovec pri Kocevski Reki, along the street to Bezgovica; 960; 14°43'41''E, 45°33'32''N; Simon Stifter; 30.6.2017

Ct83; Bosnia and Herzegovina; Republika Srpska; Drinići; 921; 16°29'41''E, 44°29'9''N; Đorđije Milanović; 01.9.2017

Ct84; Bosnia and Herzegovina; Federation of Bosnia and Herzegovina; Oštrelj, mountain Klekovača; 1130; 16°25'46''E, 44°28'25''N; Solaković S.; 28.8.2017

Ct85; Bosnia and Herzegovina; Federation of Bosnia and Herzegovina; Basanovac, mountain Grmeč; 860; 16°21'52''E, 44°40'6''N; Solaković S.; 26.8.2017

Ct86; Bosnia and Herzegovina; Federation of Bosnia and Herzegovina; Kulen vakuf, mountain Osječenica; 1080; 16°13'43''E, 44°32'15''N; Solaković S.; 29.8.2017

Ct87; Bosnia and Herzegovina; Federation of Bosnia and Herzegovina; above Mijačica, mountain Grmeč; 1090; 16°33'13''E, 44°36'9''N; Solaković S.; 27.8.2017

Ct88; Bosnia and Herzegovina; Federation of Bosnia and Herzegovina; Vodička kosa, mountain Grmeč; 1130; 16°17'53''E, 44°41'60''N; Solaković S.; 30.8.2017

Ct35; Slovakia; Žilina; Oravská vrchovina Mts, Oravský Podzámok village, Raciborská dolina valley; 550; 19°19'58''E, 49°15'32''N; Judita Kochjarová;

Ct36; Slovakia; Žilina; Oravská vrchovina Mts, between Oravský Podzámok and Pribiš villages, Pribiš brook valley; 550; 19°22'58''E, 49°15'9''N; Judita Kochjarová;

Ct37; Slovakia; Žilina; Západné Tatry Mts (Western Tatras), Oravice village, below the sedlo Bôrik saddle; 905; 19°42'33''E, 49°16'52''N; Judita Kochjarová;

Ct38; Slovakia; Žilina; Chočské vrchy Mts, Huty village, Mt Biela skala (the foot of the mountain); 940; 19°35'57''E, 49°13'22''N; Judita Kochjarová;

Ct39; Slovakia; Žilina; Západné Beskydy Mts, Oravská Polhora village, below the sedlo Hliny saddle; 780; 19°22'48''E, 49°32'35''N; Judita Kochjarová;

Ct40; Austria; Lower Austria; Baunzen; 380; 16°10'20''E, 48°11'5''N; Julian Haider; 01.5.2017

Ct49; Austria; Styria; Karnerberg N of Leutschach; 383; 15°27'18''E, 46°40'35''N; Julian Haider; 27.5.2017

Ct50; Slovenia; Ravne na Koroškem; Crna ma Koroskem; 626; 14°49'53''E, 46°27'49''N; Julian Haider; 28.5.2017

Ct51; Slovenia; Tržič; Trzic, Jelendol; 1197; 14°22'55''E, 46°24'55''N; Julian Haider; 29.5.2017

Ct52; Slovenia; Jesenice; Kranjska Gora; 903; 13°45'6''E, 46°29'14''N; Julian Haider; 30.5.2017

Ct53; Austria; Carinthia; Stockenboi, near the water fall; 899; 13°27'35''E, 46°42'13''N; Julian Haider; 31.5.2017

Ct54; Austria; Styria; Pichl; 973; 13°36'33''E, 47°23'55''N; Julian Haider; 31.5.2017

Ct55; Austria; Upper Austria; Aschau/Haiden; 490; 13°34'21''E, 47°42'57''N; Julian Haider; 01.6.2017

Ct56; Austria; Upper Austria; Steyerling; 724; 14°7'19''E, 47°49'25''N; Julian Haider; 02.6.2017

Ct57; Austria; Upper Austria; Oberplaißa; 309; 14°34'17''E, 47°52'32''N; Julian Haider; 02.6.2017

Ct58; Austria; Lower Austria; Loisbach, Mitterbergeramt; 464; 15°33'39''E, 48°31'56''N; Julian Haider; 06.6.2017

Ct41; Austria; Lower Austria; Scheuchenstein; 682; 15°59'36''E, 47°49'58''N; Julian Haider; 15.5.2017

Ct59; Germany; Bavaria; Oberwössen; 615; 12°27'16''E, 47°43'22''N; Julian Haider; 01.7.2017

Ct60; Germany; Bavaria; Miesbach; 788; 11°53'16''E, 47°46'24''N; Julian Haider; 02.7.2017

Ct61; Germany; Bavaria; Wengen; 695; 11°2'35''E, 47°55'29''N; Julian Haider; 05.7.2017

Ct62; Germany; Bavaria; Schwaigen; 697; 11°8'21''E, 47°37'11''N; Julian Haider; 03.7.2017

Ct63; Germany; Bavaria; near Zwieselberg; 871; 10°40'40''E, 47°39'14''N; Julian Haider; 04.7.2017

Ct64; Germany; Bavaria; near Oberau; 556; 13°2'29''E, 47°40'32''N; Julian Haider; 04.7.2017

Ct42; Austria; Lower Austria; Gruft, steep N-slope; 740; 15°12'24''E, 47°58'32''N; Julian Haider; 16.5.2017

Ct43; Austria; Lower Austria; Am Himmel, N-slope; 860; 15°32'20''E, 47°58'33''N; Julian Haider; 16.5.2017

Ct44; Austria; Lower Austria; Schäffern; 616; 16°7'12''E, 47°28'44''N; Julian Haider; 25.5.2017

Ct45; Austria; Styria; Mürzsteg; 890; 15°27'26''E, 47°40'23''N; Julian Haider; 26.5.2017

Ct46; Austria; Styria; Mitterbach (W of Birkfeld); 1022; 15°32'48''E, 47°22'30''N; Julian Haider; 26.5.2017

Ct47; Austria; Lower Austria; Oberdorf; 872; 15°3'47''E, 47°31'31''N; Julian Haider; 26.5.2017

Ct48; Austria; Styria; Neuhof, Gleinalpe; 912; 15°7'15''E, 47°13'32''N; Julian Haider; 27.5.2017

Ct30; Croatia; Karlovac; Klek; ; 15°8'57''E, 45°14'37''N; Ivana Rešetnik; 23.6.2017

Ct31; Croatia; Primorje-Gorski Kotar; Lokve, Golubinjak; ; 14°45'55''E, 45°21'27''N; Ivana Rešetnik; 24.6.2017

Ct27; Croatia; Krapina-Zagorje; Medvednica, Kraljičin zdenac; ; 15°56'23''E, 45°52'32''N; Ivana Rešetnik; 07.5.2017

Ct33; Croatia; Primorje-Gorski Kotar; NP Risnjak, Lazac; ; 14°35'59''E, 45°27'9''N; Ivana Rešetnik; 24.6.2017

Ct32; Croatia; Primorje-Gorski Kotar; NP Risnjak, Medvjeđa vrata; ; 14°37'23''E, 45°24'60''N; Ivana Rešetnik; 24.6.2017

Ct29; Croatia; Lika-Senj; Plitvice, Kapela Korenička-Rudanovac; ; 15°40'54''E, 44°47'51''N; Ivana Rešetnik; 22.6.2017

Ct28; Croatia; Lika-Senj; Plitvice, Poljanak; ; 15°35'56''E, 44°55'46''N; Ivana Rešetnik; 22.6.2017

Ct26; Croatia; Krapina-Zagorje; Strahinjčica, above Gorjani Sutinski; ; 15°56'20''E, 46°10'47''N; Lj. Borovečki-Voska; 03.6.2017

Ct24; Croatia; Primorje-Gorski Kotar; Učka, Vojak; ; 14°12'27''E, 45°17'59''N; I. Rešetnik, S. Bogdanović, M. Temunović; 23.7.2017

Ct34; Croatia; Lika-Senj; Velebit, Visočica; ; 15°21'31''E, 44°26'14''N; I. Rešetnik, P. Schönswetter; 28.6.2017

Ct25; Croatia; Zagreb; Žumberak, Sopotski slap; ; 15°23'49''E, 45°45'52''N; Ivana Rešetnik; 14.4.2017

Af4; Italy; Veneto; Bosconero group, Cima del'Albero, 1.5 km SSW of the summit; 1640; 12°16'6''E, 46°17'24''N; Christoph Dobeš; 02.9.2015

***Euphorbia carniolica***

Ec1; Slovenia; Gorenjska; Julian Alps, Soteska, near Sava Bohinjka, in the forest next to the bus stop; 485; 14°2'51''E, 46°18'10''N; Spela Novak; 09.8.2016

Ec2; Croatia; Primorje-Gorski Kotar; Gorski Kotar, Samarske stijene, in the vicinity of Ratkovo skloniste; ; 14°57'8''E, 45°13'33''N; Ivana Rešetnik; 03.9.2016

Ec3; Italy; Veneto; Cima Alta, 2.7 km WNW Susegana and 0.7 km NE of San Daniele-tombola; 100; 12°13'6''E, 45°51'25''N; Christoph Dobeš; 12.6.2017

Ec4; Italy; Veneto; 0.4 km SW of Fener (W Valdobbiadene); 350; 11°56'22''E, 45°54'8''N; Christoph Dobeš; 13.6.2017

Ec5; Italy; Veneto; 0.3 km NW Titele (9 km SW Agordo); 620; 11°59'16''E, 46°12'42''N; Christoph Dobeš; 14.6.2017

Ec6; Italy; Trentino; Val di Sella, localitá Dosso 3 km ENE Sella and 4 km S of Borgo Valsugana; 770; 11°26'56''E, 46°1'4''N; Christoph Dobeš; 15.6.2017

Ec7; Italy; Veneto; Altopiano die Setti Communi, 2.0 km W of San Antonio, N of the main road; 1060; 11°11'18''E, 45°45'29''N; Christoph Dobeš; 16.6.2017

Ec9; Italy; Lombardia; Lago d'Idro, 1.8 km NW of Anfo; 730; 10°29'7''E, 45°46'51''N; Christoph Dobeš; 17.6.2017

Ec8; Italy; Trentino; Brenta mountains, 1.2 km NE of Binio; 1200; 10°46'12''E, 46°4'26''N; Christoph Dobeš; 17.6.2017

Ec10; Italy; Lombardia; Spiazzi di Gromo, immediately E of the parking place (E of Boario Spiazzi); 1210; 9°57'51''E, 45°58'18''N; Christoph Dobeš; 19.6.2017

Ec11; Italy; Lombardia; 1 km S of Cremeno; 820; 9°28'20''E, 45°55'32''N; Christoph Dobeš; 20.6.2017

Ec65; Ukraine; Ivano-Frankivska oblast; 4 km E of Mykulychyn; 910; 24°38'46''E, 48°25'6''N; Simon Stifter; 11.6.2017

Ec49; Romania; Gorj; forest between Tismana and Pestisani; 290; 22°59'55''E, 45°3'9''N; Simon Stifter; 08.8.2017

Ec66; Ukraine; Ivano-Frankivska oblast; small valley 5 km NW of Tatariv; 880; 24°30'28''E, 48°22'41''N; Simon Stifter; 11.6.2017

Ec50; Romania; Caraș-Severin; 3 km SE of Borlova along the river; 600; 22°24'36''E, 45°20'7''N; Simon Stifter; 10.8.2017

Ec61; Slovenia; Tolmin; 5 km after Borjana into the Nadiza Valley, beside the street; 300; 13°26'41''E, 46°14'2''N; Simon Stifter; 24.5.2017

Ec67; Ukraine; Ivano-Frankivska oblast; 0.3 km S from the parking place of Mount Hoverla (Vorokhta); 1282; 24°32'10''E, 48°9'39''N; Simon Stifter; 12.6.2017

Ec51; Romania; Hunedoara; on the N side of the Lacul gura apelor lake; 1150; 22°42'38''E, 45°20'3''N; Simon Stifter; 10.8.2017

Ec52; Slovenia; Tolmin; Klavze, 0.25 km S from baca river; 275; 13°49'4''E, 46°9'23''N; Simon Stifter; 26.5.2017

Ec62; Ukraine; Ivano-Frankivska oblast; 0.5 km S of Burkut, along the river; 944; 24°41'49''E, 47°56'17''N; Simon Stifter; 12.6.2017

Ec31; Romania; Satu Mare; between Barsau and Certeju de Sus, close to the two lakes on the E; 350; 22°57'0''E, 45°57'25''N; Simon Stifter; 10.8.2017

Ec53; Slovenia; Škofja Loka; E of Praprotno get over the river, take road on the left for 0.3 km, forest on E; 446; 14°15'55''E, 46°11'50''N; Simon Stifter; 26.5.2017

Ec63; Ukraine; Chernivetska oblast; 3 km SW of Nyzhnii Yalovets, along the street; 887; 24°59'9''E, 47°50'12''N; Simon Stifter; 13.6.2017

Ec32; Romania; Arad; Milova Valley; 550; 21°49'1''E, 46°9'5''N; Simon Stifter; 11.8.2017

Ec54; Slovenia; Cerknica; on Mt. Slivnica (Cerknica) behind the Hut: Dom na Slivnici; 1093; 14°24'22''E, 45°47'22''N; Simon Stifter; 27.5.2017

Ec64; Ukraine; Chernivetska oblast; 5 km N of Ruska, along the mountain street; 900; 25°13'43''E, 47°56'12''N; Simon Stifter; 13.6.2017

Ec33; Romania; Arad; 5 km into the Valley SE of Iacobini, along the river; 330; 22°23'23''E, 46°11'54''N; Simon Stifter; 11.8.2017

Ec55; Slovenia; Ptuj; 2 km S of Kozminci; 300; 15°51'17''E, 46°16'56''N; Simon Stifter; 27.6.2017

Ec34; Romania; Caraș-Severin; 2 km NE of Rachita (Sebes); 400; 23°31'59''E, 45°53'36''N; Simon Stifter; 12.8.2017

Ec56; Slovenia; Žalec; close to Jama Pekel cave ; 350; 15°7'58''E, 46°17'24''N; Simon Stifter; 28.6.2017

Ec35; Romania; Sibiu; going from Sadu to Rau Sadului, after 5km take road on the left and follow until a small river (1,5km); 660; 24°9'18''E, 45°38'26''N; Simon Stifter; 12.8.2017

Ec57; Slovenia; Šmarje pri Jelšah; 2 km SW of Trebce take a small street which brings you to a farm S of Bistrica river; 300; 15°37'26''E, 46°1'59''N; Simon Stifter; 29.6.2017

Ec36; Romania; Argeș; E of Sativ (Podu Dambovitei); 860; 25°11'29''E, 45°25'45''N; Simon Stifter; 14.8.2017

Ec58; Slovenia; Črnomelj; from Crmosnjece go to Komarna Vas Farm, 1 km NW; 630; 15°4'5''E, 45°40'55''N; Simon Stifter; 29.6.2017

Ec37; Romania; Brașov; 2 km E of Budila (Brasov); 650; 25°50'47''E, 45°39'56''N; Simon Stifter; 15.8.2017

Ec59; Slovenia; Kočevje; 1.5 km S of Borovec pri Kocevski Reki; 800; 14°46'52''E, 45°32'12''N; Simon Stifter; 30.6.2017

Ec38; Romania; Covasna; at the end of the street in Estelnic; 680; 26°12'28''E, 46°7'5''N; Simon Stifter; 15.8.2017

Ec60; Slovenia; Domžale; 0.5 km W of Cesnjice pri Moravcah; 380; 14°44'13''E, 46°7'29''N; Simon Stifter; 30.6.2017

Ec39; Romania; Covasna; 1 km S of Malnas (Sfantu Gheorghe); 600; 25°50'51''E, 45°59'57''N; Simon Stifter; 16.8.2017

Ec40; Romania; Brașov; pass W of Maierus, at the first turn; 620; 25°26'35''E, 45°53'30''N; Simon Stifter; 16.8.2017

Ec41; Romania; Alba; W of Baia de Aries, 0.5 km N of the street; 560; 23°16'8''E, 46°22'56''N; Simon Stifter; 17.8.2017

Ec42; Romania; Alba; starting point of the way to Mt. Muntisorul (Garda de Sus), S of river; 840; 22°47'53''E, 46°27'25''N; Simon Stifter; 18.8.2017

Ec43; Romania; Maramureș; Costitui: forest behind castelul Apafi; 470; 24°1'31''E, 47°52'36''N; Simon Stifter; 20.8.2017

Ec44; Romania; Bistrița-Năsăud; along the street S of Socel to Dealu Stefanitei; 650; 24°25'43''E, 47°36'37''N; Simon Stifter; 20.8.2017

Ec45; Romania; Bistrița-Năsăud; S of Nasaud, after the bridge take the small road on the right and follow for 2 km; 380; 24°23'58''E, 47°16'17''N; Simon Stifter; 21.8.2017

Ec46; Romania; Cluj; from Rugasesti take the street to Salatruc, after 0.5 km on left hand side; 350; 23°51'55''E, 47°14'50''N; Simon Stifter; 21.8.2017

Ec47; Romania; Mureș; from Lunca Bradului 2 km into Ilva Valley; 700; 25°6'58''E, 46°58'47''N; Simon Stifter; 21.8.2017

Ec48; Romania; Harghita; on Lacu Rosu pass, along the river; 1050; 25°47'50''E, 46°47'37''N; Simon Stifter; 22.8.2017

Ec68; Bosnia and Herzegovina; Federation of Bosnia and Herzegovina; Ljuta; 900; 18°18'8''E, 43°34'30''N; Bogunić Faruk; 12.7.2017

Ec29; Serbia; Mačvanski okrug; Jablanik, Markovića brdo; 1120; 19°38'55''E, 44°10'48''N; Marjan Niketić; 30.7.2017

Ec69; Bosnia and Herzegovina; Federation of Bosnia and Herzegovina; Stipića livade, mountain Čvrsnica; 1370; 17°38'2''E, 43°40'18''N; Bogunić Faruk; 15.7.2017

Ec30; Serbia; Zlatiborski okrug; Tara, Predov krst; 1045; 19°18'13''E, 43°56'25''N; Nevena Kuzmanović, Tijana Milekić; 22.8.2017

Ec70; Bosnia and Herzegovina; Federation of Bosnia and Herzegovina; Bijambare; 980; 18°30'3''E, 44°4'10''N; Bogunić Faruk; 26.7.2017

Ec71; Bosnia and Herzegovina; Federation of Bosnia and Herzegovina; Ravna vala, mountain Igman; 1300; 18°15'54''E, 43°44'17''N; Bogunić Faruk; 16.5.2017

Ec72; Bosnia and Herzegovina; Federation of Bosnia and Herzegovina; Basanovac, mountain Grmeč; 860; 16°21'52''E, 44°40'6''N; Bogunić Faruk; 26.8.2017

Ec73; Bosnia and Herzegovina; Republika Srpska; Drinići; 943; 16°28'11''E, 44°29'32''N; Đorđije Milanović; 01.9.2017

Ec74; Montenegro; Plužine; Medudo near Pivsko lake and Plužine; 995; 18°49'6''E, 43°11'18''N; Đorđije Milanović; 21.6.2017

Ec75; Bosnia and Herzegovina; Republika Srpska; Jelova Dola, mountain Maglić; 1602; 18°43'14''E, 43°16'34''N; Đorđije Milanović; 09.8.2017

Ec76; Bosnia and Herzegovina; Federation of Bosnia and Herzegovina; Kulen vakuf, mountain Osječenica; 1080; 16°13'43''E, 44°32'15''N; Solaković S.; 29.8.2017

Ec27; Austria; Carinthia; Trögnerbach S of the gorge; 903; 14°29'7''E, 46°27'7''N; Julian Haider; 30.5.2017

Ec28; Slovenia; Radovljica; Mesnovec; 1283; 13°56'56''E, 46°20'55''N; Julian Haider; 31.5.2017

Ec25; Croatia; Karlovac; Generalski Stol; ; 15°22'47''E, 45°21'12''N; D. Škrtić; 18.6.2017

Ec12; Croatia; Karlovac; Klek; ; 15°8'50''E, 45°14'59''N; Ivana Rešetnik; 23.6.2017

Ec18; Croatia; Primorje-Gorski Kotar; Lokve, Golubinjak; ; 14°45'56''E, 45°21'26''N; Ivana Rešetnik; 24.6.2017

Ec19; Croatia; Primorje-Gorski Kotar; Mala Lešnica; ; 14°51'8''E, 45°26'14''N; Ivana Rešetnik; 23.6.2017

Ec21; Croatia; Primorje-Gorski Kotar; NP Risnjak, Lazac; ; 14°35'59''E, 45°27'8''N; Ivana Rešetnik; 24.6.2017

Ec20; Croatia; Primorje-Gorski Kotar; NP Risnjak, Malo Selo; ; 14°42'2''E, 45°25'54''N; Ivana Rešetnik; 24.6.2017

Ec22; Croatia; Virovitica-Podravina; Papuk, Jankovac; ; 17°41'31''E, 45°30'51''N; I. Rešetnik, M. Doboš; 29.4.2017

Ec26; Croatia; Pozega-Slavonia; Papuk, Klinovac; ; 17°38'57''E, 45°30'43''N; I. Rešetnik, M. Doboš; 29.4.2017

Ec14; Croatia; Virovitica-Podravina; Papuk, peak Ponori above Military road; ; 17°37'40''E, 45°31'6''N; I. Rešetnik, M. Doboš; 29.4.2017

Ec15; Croatia; Lika-Senj; Plitvice, Gornji Babin Potok-Končarev kraj; ; 15°33'18''E, 44°49'39''N; Ivana Rešetnik; 22.6.2017

Ec17; Croatia; Lika-Senj; Plitvice, Kapela Korenička-Rudanovac; ; 15°40'60''E, 44°47'49''N; Ivana Rešetnik; 22.6.2017

Ec13; Croatia; Zagreb; Samoborsko gorje, Žužići; ; 15°39'1''E, 45°46'43''N; Ivana Rešetnik; 14.4.2017

Ec24; Croatia; Krapina-Zagorje; Strahinjčica, Radoboj; ; 15°56'15''E, 46°11'12''N; Ivana Rešetnik; 13.5.2017

Ec16; Croatia; Lika-Senj; Velebit, Lomska duliba; ; 15°0'57''E, 44°46'35''N; I. Rešetnik, P. Schönswetter; 27.6.2017

Ec23; Croatia; Zagreb; Žumberak, Sopotski slap; ; 15°23'49''E, 45°45'52''N; Ivana Rešetnik; 14.4.2017

***Hacquetia epipactis***

He1; Slovenia; Gorenjska; Karavanke mountains, between the mountain hut Valvasorjev dom and the pasture Žirovniška planina; 1220; 14°9'53''E, 46°25'36''N; Spela Novak; 03.8.2016

He2; Slovenia; Gorenjska; Kamniško-Savinjske Alpe, 0.01 km S of the big parking place in Ravenska Kočna; 1174; 14°32'9''E, 46°22'59''N; Spela Novak; 07.8.2016

He3; Slovenia; Gorenjska; Kamniško-Savinjske Alpe, Potoče, 0.5 km NW of Javorov vrh; 1320; 14°27'37''E, 46°19'38''N; Spela Novak; 15.9.2016

He4; Slovenia; Gorenjska; Kamniško-Savinjske Alpe, slope of Kriška gora, Zgornje Vetrno, 0.050 km E of the parking place for "strma pot na Kriško goro"; 850; 14°19'6''E, 46°20'39''N; Spela Novak; 30.10.2016

He5; Croatia; Karlovac; Generalski Stol; ; 15°22'47''E, 45°21'12''N; D. Škrtić; 18.6.2017

He40; Czech Republic; Olomouc; Olomouc, Grygov; ; 17°18'36''E, 49°31'11''N; Bohumil Mandák;

He49; Czech Republic; Moravia-Silesia; Velké Doly; ; 18°37'59''E, 49°42'53''N; Bohumil Mandák;

He50; Czech Republic; Moravia-Silesia; Trnávka; ; 18°11'1''E, 49°40'54''N; Bohumil Mandák;

He41; Czech Republic; Zlín; Halenkovice; ; 17°26'41''E, 49°11'11''N; Bohumil Mandák;

He42; Czech Republic; Zlín; Uherský Brod; ; 17°39'31''E, 49°3'10''N; Bohumil Mandák;

He43; Czech Republic; South Moravia; Velká nad Veličkou; ; 17°33'41''E, 48°52'28''N; Bohumil Mandák;

He44; Czech Republic; Zlín; Kunkovice; ; 17°12'26''E, 49°10'12''N; Bohumil Mandák;

He45; Czech Republic; South Moravia; Věteřov; ; 17°3'44''E, 49°1'30''N; Bohumil Mandák;

He46; Czech Republic; South Moravia; Ždánice; ; 17°1'9''E, 49°5'46''N; Bohumil Mandák;

He47; Czech Republic; South Moravia; Zdravý Voda; ; 16°56'51''E, 49°4'15''N; Bohumil Mandák;

He48; Czech Republic; Plzeň; Dražovice; 394; 16°56'45''E, 49°11'3''N; Bohumil Mandák;

He38; Slovenia; Nova Gorica; 1 km W of Vogrsko on Highway H4, go 0.5 km N; 100; 13°41'6''E, 45°55'1''N; Simon Stifter; 23.5.2017

He27; Poland; Opole (Upper Silesia); 2 km E of Rozumice; 270; 17°59'39''E, 50°1'12''N; Simon Stifter; 01.6.2017

He39; Slovenia; Tolmin; 4 km SE of Idrsko, beside the street; 180; 13°36'53''E, 46°12'51''N; Simon Stifter; 25.5.2017

He28; Poland; Silesia; N of Cieszyn, close to the border, 2 km before Pogwizdow; 260; 18°36'26''E, 49°46'43''N; Simon Stifter; 02.6.2017

He30; Slovenia; Tolmin; Klavze, 0.25 km S from baca river; 273; 13°48'50''E, 46°9'30''N; Simon Stifter; 26.5.2017

He29; Poland; Lesser Poland; 2 km NE of Mogilany; 350; 19°52'52''E, 49°56'52''N; Simon Stifter; 02.6.2017

He31; Slovenia; Škofja Loka; E of Praprotno get over the river, take road on the left for 0.3 km, forest on E; 446; 14°15'55''E, 46°11'50''N; Simon Stifter; 26.5.2017

He32; Slovenia; Cerknica; on Mt. Slivnica (Cerknica) behind the Hut: Dom na Slivnici; 1093; 14°24'22''E, 45°47'22''N; Simon Stifter; 27.5.2017

He33; Slovenia; Slovenska Bistrica; from Zgornje Polycane take the street to Na Boc until a large meadow (2 km); 700; 15°35'17''E, 46°16'56''N; Simon Stifter; 28.6.2017

He34; Slovenia; Šmarje pri Jelšah; 2 km SW of Trebce take a small street which brings you to a farm S of Bistrica river; 300; 15°37'26''E, 46°1'59''N; Simon Stifter; 29.6.2017

He35; Slovenia; Črnomelj; from Crmosnjece go to Komarna Vas Farm, 1 km NW; 630; 15°4'5''E, 45°40'55''N; Simon Stifter; 29.6.2017

He36; Slovenia; Kočevje; 1.5 km S of Borovec pri Kocevski Reki; 800; 14°46'52''E, 45°32'12''N; Simon Stifter; 30.6.2017

He37; Slovenia; Domžale; 0.5 km W of Cesnjice pri Moravcah; 380; 14°44'13''E, 46°7'29''N; Simon Stifter; 30.6.2017

He51; Bosnia and Herzegovina; Federation of Bosnia and Herzegovina; botanical garden Sarajevo; ; °'0''E, °'0''N; Bogunić Faruk; 08.8.2017

He19; Slovakia; Žilina; Veľká Fatra Mts, Blatnica village, Blatnická dolina valley; 700; 18°57'1''E, 48°54'47''N; Judita Kochjarová; 02.7.2017

He20; Slovakia; Žilina; Veľká Fatra Mts, Ľubochňa village, Ľubochnianska dolina valley; 480; 19°9'17''E, 49°6'49''N; Judita Kochjarová; 03.7.2017

He21; Slovakia; Žilina; Malá Fatra-Žiar Mts, Kláštor pod Znievom village, Mt Zniev (the foot of the mountain); 560; 18°47'15''E, 48°58'14''N; Judita Kochjarová;

He22; Slovakia; Žilina; Kysucká vrchovina Mts, Mt Veľké Ostré between the villages Radoľa and Lopušné Pažite; 505; 18°48'29''E, 49°16'48''N; Judita Kochjarová;

He23; Slovakia; Žilina; Turčianska kotlina basin, Sučany village, Mt Bukovina S from the village; 526; 18°59'42''E, 49°5'10''N; Judita Kochjarová;

He16; Slovakia; Banska Bystrica; Zvolenská kotlina basin, between Banská Bystrica town and Horná Mičiná village; 447; 19°10'34''E, 48°42'56''N; R. Hrivnák; 29.6.2017

He24; Austria; Carinthia; Lavantal, Burgstallkogel; 529; 14°57'13''E, 46°38'40''N; Julian Haider; 28.5.2017

He17; Slovakia; Trenčin; Nitrianska pahorkatina Mts, Látkovce village, NNE from the village; 298; 18°21'53''E, 48°43'8''N; R. Hrivnák; 29.6.2017

He25; Austria; Carinthia; Bad Eisenkappel; 640; 14°35'30''E, 46°30'3''N; Julian Haider; 28.5.2017

He18; Slovakia; Trenčin; Strážovské vrchy Mts, Horné Motešice village, N from the village; 313; 18°10'55''E, 48°51'6''N; R. Hrivnák; 29.6.2017

He26; Slovenia; Tržič; Trzic; 806; 14°20'20''E, 46°23'52''N; Julian Haider; 29.5.2017

He15; Croatia; Karlovac; Klek, Soviljica; ; 15°9'38''E, 45°14'56''N; Ivana Rešetnik; 23.6.2017

He6; Croatia; Primorje-Gorski Kotar; Mala Lešnica; ; 14°51'8''E, 45°26'15''N; Ivana Rešetnik; 23.6.2017

He8; Croatia; Krapina-Zagorje; Medvednica, Glavica; ; 15°53'9''E, 45°51'2''N; Ivana Rešetnik; 09.4.2017

He10; Croatia; Primorje-Gorski Kotar; NP Risnjak, Lazac; ; 14°35'59''E, 45°27'9''N; Ivana Rešetnik; 24.6.2017

He11; Croatia; Primorje-Gorski Kotar; NP Risnjak, Malo Selo; ; 14°42'2''E, 45°25'54''N; Ivana Rešetnik; 24.6.2017

He14; Croatia; Lika-Senj; Plitvice, Kapela Korenička-Rudanovac; ; 15°40'60''E, 44°47'49''N; Ivana Rešetnik; 22.6.2017

He13; Croatia; Zagreb; Samoborsko gorje, Žužići; ; 15°39'1''E, 45°46'43''N; Ivana Rešetnik; 14.4.2017

He7; Croatia; Krapina-Zagorje; Strahinjčica, above Gorjani Sutinski; ; 15°55'56''E, 46°10'50''N; Lj. Borovečki-Voska; 03.6.2017

He9; Croatia; Krapina-Zagorje; Strahinjčica, Radoboj; ; 15°56'20''E, 46°10'56''N; Ivana Rešetnik; 13.5.2017

He12; Croatia; Zagreb; Žumberak, Sopotski slap; ; 15°23'49''E, 45°45'52''N; Ivana Rešetnik; 14.4.2017

***Helleborus niger***

Hn1; Slovenia; Gorenjska; Karavanke mountains, between the mountain hut Valvasorjev dom and the pasture Žirovniška planina; 1223; 14°9'26''E, 46°25'28''N; Spela Novak; 03.8.2016

Hn3; Austria; Lower Austria; Northern Calcareous Alps, Dürrenstein, Seetal, E of the Mittersee, left and right of the service road; 770; 15°4'35''E, 47°49'40''N; Christoph Dobeš; 27.8.2016

Hn2; Slovenia; Gorenjska; Kamniško-Savinjske Alpe, Potoče, 0.5 km NW of Javorov vrh; 1320; 14°27'37''E, 46°19'38''N; Spela Novak; 15.9.2016

Hn4; Slovenia; Gorenjska; Kamniško-Savinjske Alpe, Logarska dolina, 0.1 km E of the hut Koča na Klemenči jami; 1220; 14°38'7''E, 46°22'43''N; Spela Novak; 04.11.2016

Hn5; Italy; Friuli-Venezia Giulia; Alpi Giulie, immediately N of the village Riofreddo; 860; 13°34'5''E, 46°28'17''N; Christoph Dobeš; 09.6.2017

Hn6; Italy; Friuli-Venezia Giulia; 0.8 km WSW of Studena Alta; 880; 13°15'57''E, 46°30'22''N; Christoph Dobeš; 11.6.2017

Hn7; Italy; Veneto; 6.2 km S of Seren del Grappa, Misola; 730; 11°50'3''E, 45°56'4''N; Christoph Dobeš; 13.6.2017

Hn8; Italy; Veneto; Altopiano dei Setti Communi, 0.7 km NE of Mezzaselva; 1030; 11°26'12''E, 45°52'27''N; Christoph Dobeš; 15.6.2017

Hn9; Italy; Veneto; Altopiano die Setti Communi, 2.0 km W of San Antonio, N of the main road; 1060; 11°11'18''E, 45°45'29''N; Christoph Dobeš; 16.6.2017

Hn10; Italy; Trentino; Brenta mountains, 1.2 km NE of Binio; 1200; 10°46'12''E, 46°4'26''N; Christoph Dobeš; 17.6.2017

Hn11; Italy; Lombardia; Lago d'Idro, 0.9 km NW of Anfo; 580; 10°29'12''E, 45°46'17''N; Christoph Dobeš; 17.6.2017

Hn12; Italy; Lombardia; 1.3 km SE of Albino and 2 km NE of Pradalunga; 410; 9°48'33''E, 45°45'14''N; Christoph Dobeš; 19.6.2017

Hn13; Italy; Lombardia; Spiazzi di Gromo, immediately E of the parking place (E of Boario Spiazzi); 1210; 9°57'51''E, 45°58'18''N; Christoph Dobeš; 19.6.2017

Hn14; Italy; Lombardia; Val di Faggio, 0.5 km NE of Moggio; 890; 9°29'47''E, 45°55'54''N; Christoph Dobeš; 20.6.2017

Hn16; Switzerland; Ticino; 2.1 km E of Cadro, Alpe Bolla; 1170; 9°0'39''E, 46°2'20''N; Christoph Dobeš; 21.6.2017

Hn15; Switzerland; Ticino; Lago di Lugano, peninsula S of Calsano; 460; 8°52'44''E, 45°57'59''N; Christoph Dobeš; 22.6.2017

Hn48; Italy; Lombardia; Lago d’Iseo, Monte Isola, along the trail leading from Peschiera Maraglio to the Santuario Madonna della Ceriola; 340; 10°5'21''E, 45°41'45''N; Christoph Dobeš; 28.10.2017

Hn49; Austria; Styria; Weißwände S of the Allachhütte, ca. 5 km E of Turrach; 1800; 13°56'49''E, 46°58'8''N; Th. Zimmermann; 25.7.2019

Hn53; Austria; Tyrol; Karwendel, Kranebitter Klamm, above the narrows of the gorge; 880; 11°19'39''E, 47°16'40''N; Peter Schönswetter; 14.9.2019

Hn54; Austria; Tyrol; Karwendel, above of Höttinger Bild; 1060; 11°21'57''E, 47°17'0''N; Peter Schönswetter; 15.9.2019

Hn23; Croatia; Primorje-Gorski Kotar; Donji Ložac; ; 14°52'30''E, 45°26'47''N; Ivana Rešetnik; 23.6.2017

Hn50; Italy; Friuli-Venezia Giulia; Tramonti di Sopra, along the path to Forcella Rovin; 720; 12°48'35''E, 46°20'34''N; Simon Stifter; 30.8.2019

Hn43; Slovenia; Tolmin; from Soca bridge W of Tolmin, 0.1 km N; 160; 13°43'2''E, 46°11'5''N; Simon Stifter; 25.5.2017

Hn51; Italy; Friuli-Venezia Giulia; Barcis, localita Predaia ; 460; 12°33'7''E, 46°12'35''N; Simon Stifter; 31.8.2019

Hn44; Slovenia; Škofja Loka; E of Praprotno get over the river, take road on the left for 0.3 km, forest on E; 570; 14°16'13''E, 46°11'47''N; Simon Stifter; 26.5.2017

Hn52; Italy; Friuli-Venezia Giulia; first turn of the street from San Pietro to the Monte Ragogna; 300; 12°58'12''E, 46°12'31''N; Simon Stifter; 31.8.2019

Hn45; Slovenia; Cerknica; on the way to Mt. Slivnica, 1km after Cerknica; 660; 14°22'36''E, 45°48'10''N; Simon Stifter; 27.5.2017

Hn46; Slovenia; Ajdovščina; from Col go to Zagolic and follow the street for one more km; 737; 13°59'17''E, 45°53'39''N; Simon Stifter; 27.5.2017

Hn47; Slovenia; Šmarje pri Jelšah; 2 km SW of Trebce take a small street which brings you to a farm S of Bistrica river; 300; 15°37'26''E, 46°1'59''N; Simon Stifter; 29.6.2017

Hn42; Slovenia; Črnomelj; from Crmosnjece go to Komarna Vas Farm, 1 km NW; 630; 15°4'5''E, 45°40'55''N; Simon Stifter; 29.6.2017

Hn28; Austria; Lower Austria; Pernitz, at "Die Brunst"; 910; 15°48'42''E, 47°52'42''N; Julian Haider; 15.5.2017

Hn37; Austria; Styria; Pichl; 973; 13°36'33''E, 47°23'55''N; Julian Haider; 31.5.2017

Hn38; Austria; Upper Austria; Aschau, above Ischl; 519; 13°33'6''E, 47°42'53''N; Julian Haider; 01.6.2017

Hn39; Austria; Upper Austria; Steyerling; 724; 14°7'19''E, 47°49'25''N; Julian Haider; 02.6.2017

Hn40; Austria; Upper Austria; Weyer, gorge leading to the river Enns; 420; 14°36'38''E, 47°52'12''N; Julian Haider; 02.6.2017

Hn41; Austria; Tyrol; Kufstein, next to Kaiserbach; 559; 12°11'13''E, 47°35'19''N; Julian Haider; 01.7.2017

Hn29; Austria; Lower Austria; St. Anton an der Jeßnitz; 645; 15°12'11''E, 47°58'33''N; Julian Haider; 16.5.2017

Hn30; Austria; Lower Austria; Moosbach, ca. 1.5 km from Am Himmel; 523; 15°31'53''E, 47°57'38''N; Julian Haider; 16.5.2017

Hn31; Austria; Styria; Mürzsteg; 890; 15°27'26''E, 47°40'22''N; Julian Haider; 26.5.2017

Hn32; Austria; Lower Austria; Oberdorf; 830; 15°3'54''E, 47°31'43''N; Julian Haider; 26.5.2017

Hn33; Slovenia; Tržič; Karawank nahe Crna; 798; 14°47'14''E, 46°28'14''N; Julian Haider; 28.5.2017

Hn34; Slovenia; Tržič; Trzic; 806; 14°20'20''E, 46°23'52''N; Julian Haider; 29.5.2017

Hn35; Slovenia; Jesenice; Kranjska Gora; 840; 13°46'52''E, 46°28'30''N; Julian Haider; 30.5.2017

Hn36; Austria; Carinthia; Mösel; 978; 13°24'60''E, 46°42'20''N; Julian Haider; 31.5.2017

Hn17; Croatia; Karlovac; Klek, Soviljica; ; 15°9'51''E, 45°15'5''N; Ivana Rešetnik; 23.6.2017

Hn24; Croatia; Primorje-Gorski Kotar; Kuželj, V. Belica valley; ; 14°46'2''E, 45°29'11''N; Ivana Rešetnik; 23.6.2017

Hn20; Croatia; Primorje-Gorski Kotar; NP Risnjak, Javornica; ; 14°33'9''E, 45°27'45''N; Ivana Rešetnik; 24.6.2017

Hn26; Croatia; Primorje-Gorski Kotar; NP Risnjak, Malo Selo; ; 14°37'22''E, 45°24'54''N; Ivana Rešetnik; 24.6.2017

Hn18; Croatia; Primorje-Gorski Kotar; NP Risnjak, Medvjeđa vrata; ; 14°37'22''E, 45°24'54''N; Ivana Rešetnik; 24.6.2017

Hn25; Croatia; Karlovac; Ogulin, Otok Oštarijski, Veljun; ; 15°14'41''E, 45°13'15''N; Ivana Rešetnik; 23.6.2017

Hn21; Croatia; Lika-Senj; Plitvice, Gornji Babin Potok - Plitvički Ljeskovac; ; 15°33'17''E, 44°49'50''N; Ivana Rešetnik; 22.6.2017

Hn22; Croatia; Lika-Senj; Plitvice, Sertić Poljana; ; 15°33'18''E, 44°55'46''N; Ivana Rešetnik; 22.6.2017

Hn19; Croatia; Zagreb; Samoborsko gorje, Žužići; ; 15°39'1''E, 45°46'43''N; Ivana Rešetnik; 14.4.2017

Hn27; Croatia; Zagreb; Žumberak, Sopotski slap; ; 15°23'49''E, 45°45'52''N; Ivana Rešetnik; 14.4.2017


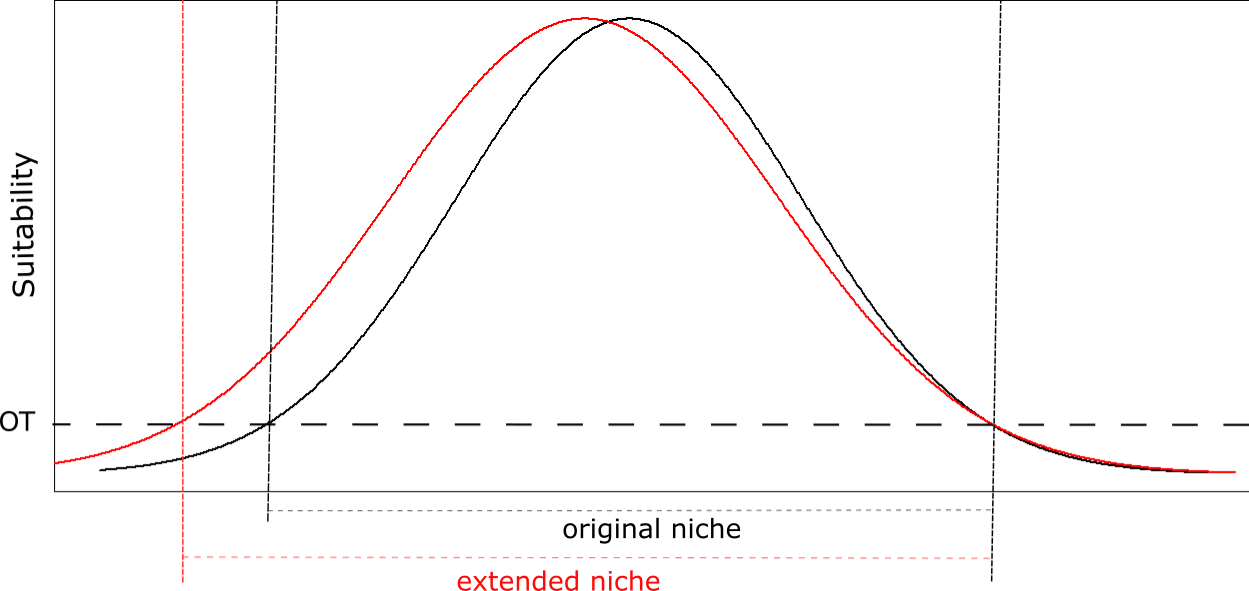


**Figure S3.** Schematic depiction of niche breadth extension. Suitability is the occurrence probability of the species along a gradient. The occurrence threshold OT is the probability above which sites are considered as suitable for the species. Black curve: original suitability. Red curve: suitability corresponding to an extended niche.


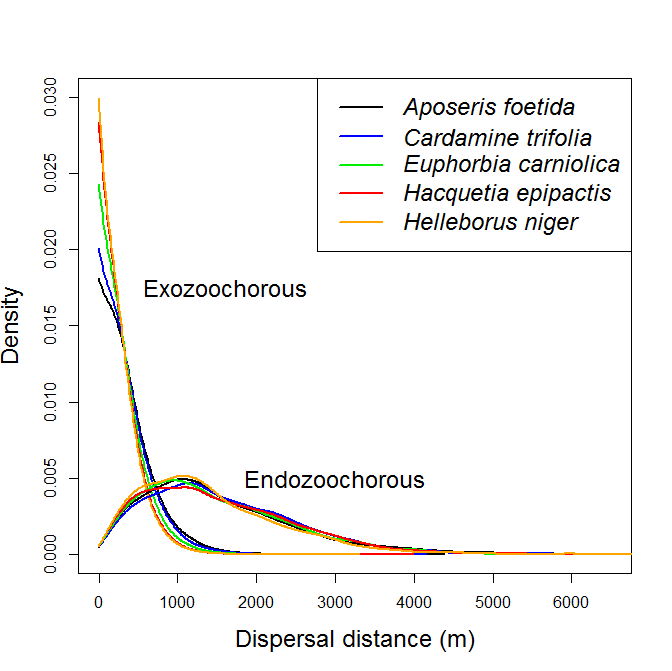


**Figure S4.** Zoochorous kernels of the five study species derived from random walk simulations of a “general large mammalian seed dispersal vector” following the procedure described in Dullinger et al. (2015).

**Appendix S5**

Results of continuous phylogeographic diffusion analysis using relaxed random walks of the collected genetic data of the species.

-> zip-file with one kml-file for each species

Af: *Aposeris foetida*

Ct: *Cardamine trifolia*

Ec: *Euphorbia carniolica*

He: *Hacquetia epipactis*

Hn: *Helleborus niger*

**Appendix S6. Sensitivity test for the sufficiency of 10 replicates per setting.**

To test whether ten replicated model runs using the same setting of model parameters were sufficient to identify the setting with the best match between mapped and predicted current distribution, we randomly selected 5, 6, 7, 8 and 9 replicates (out of the ten replicates available) for each setting of each species and identified the best setting (i.e., the one with the highest TSS). This selection and identification of the best setting was repeated 100 times for each number of replicates.

Using six replicates, the best overall setting was correctly identified in >50% of the repeated selections. The proportion of correct identifications increased to >85% when using nine replicates (Table S6). We conclude that the likelihood to identify the best setting as such is sufficiently high when using ten replicates.

**Table S6.** Proportion of repeated selections based on a reduced number of replicates revealing the same best setting as using the full set of ten replicates. A value of 100 means that all repeated selections resulted in the same best setting. NBE - niche breadth extension; SZE - standard zoochorous dispersal; LDD – Long-distance dispersal. Low and high demographic rates are given separately, as the parameters SZD and demography are antagonistic (see Results in the main text).

|  | Setting | | | | Number of replicates | | | | |
| --- | --- | --- | --- | --- | --- | --- | --- | --- | --- |
|  | Demography | NBE (%) | SZD | LDD | 5 | 6 | 7 | 8 | 9 |
| *Aposeris foetida* | high | 20 | 5∙10^-8^ | 10^-7^ | 96 | 100 | 100 | 100 | 100 |
|  | low | 20 | 5∙10^-7^ | 10^-7^ | 81 | 84 | 97 | 96 | 100 |
| in restricted area | high | 20 | 5∙10^-8^ | 10^-7^ | 62 | 52 | 77 | 81 | 90 |
|  | low | 10 | 5∙10^-8^ | 10^-7^ | 47 | 56 | 67 | 84 | 86 |
| *Cardamine trifolia* | high | 20 | 5∙10^-8^ | 10^-7^ | 99 | 98 | 100 | 100 | 100 |
|  | low | 20 | 5∙10^-6^ | 10^-7^ | 63 | 72 | 81 | 78 | 88 |
| *Euphorbia carniolica* | high | 20 | 5∙10^-7^ | 0 | 68 | 76 | 77 | 77 | 93 |
|  | low | 20 | 5∙10^-5^ | 10^-7^ | 99 | 99 | 100 | 100 | 100 |
| *Hacquetia epipactis* | high | 20 | 5∙10^-7^ | 10^-7^ | 85 | 85 | 92 | 98 | 100 |
|  | low | 20 | 5∙10^-6^ | 10^-7^ | 53 | 68 | 61 | 80 | 95 |
| *Helleborus niger* | high | 20 | 5∙10^-8^ | 10^-7^ | 48 | 64 | 60 | 80 | 95 |
|  | low | 20 | 5∙10^-6^ | 10^-7^ | 100 | 100 | 100 | 100 | 100 |


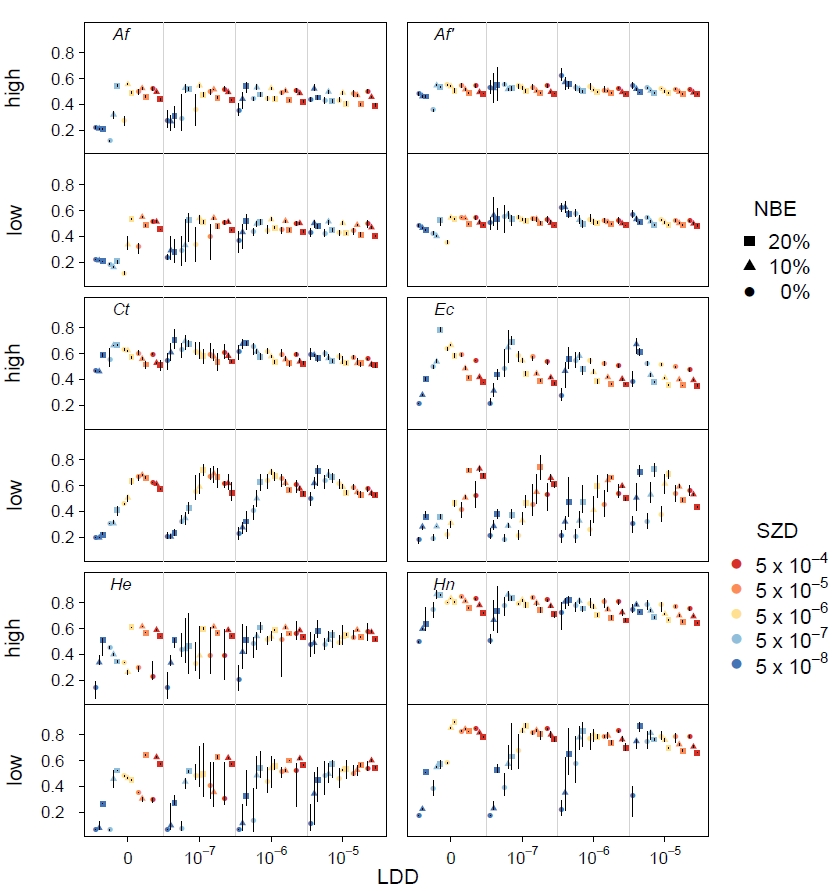


**Figure S13.** Match between the mapped current distribution of species and the current distributions predicted by model runs varying in the parameter settings for niche breadth extension (NBE), standard zoochorous dispersal (SZD) and long-distance dispersal (LDD) evaluated as the True Skills Statistic (TSS). Demographic rates were assumed to be at the upper (“high”) and lower (“low”) end of a plausible range of values. Symbols represent the average TSS values among ten replicates with the same parameter setting for each species, and vertical lines show the range of TSS values. Af: *Aposeris foetida*, Af´: *Aposeris foetida* evaluated within a restricted area excluding the Carpathians (see Figure 4 in main text), Ct: *Cardamine trifolia*, Ec: *Euphorbia carniolica*, He: *Hacquetia epipactis*, Hn: *Helleborus niger*.


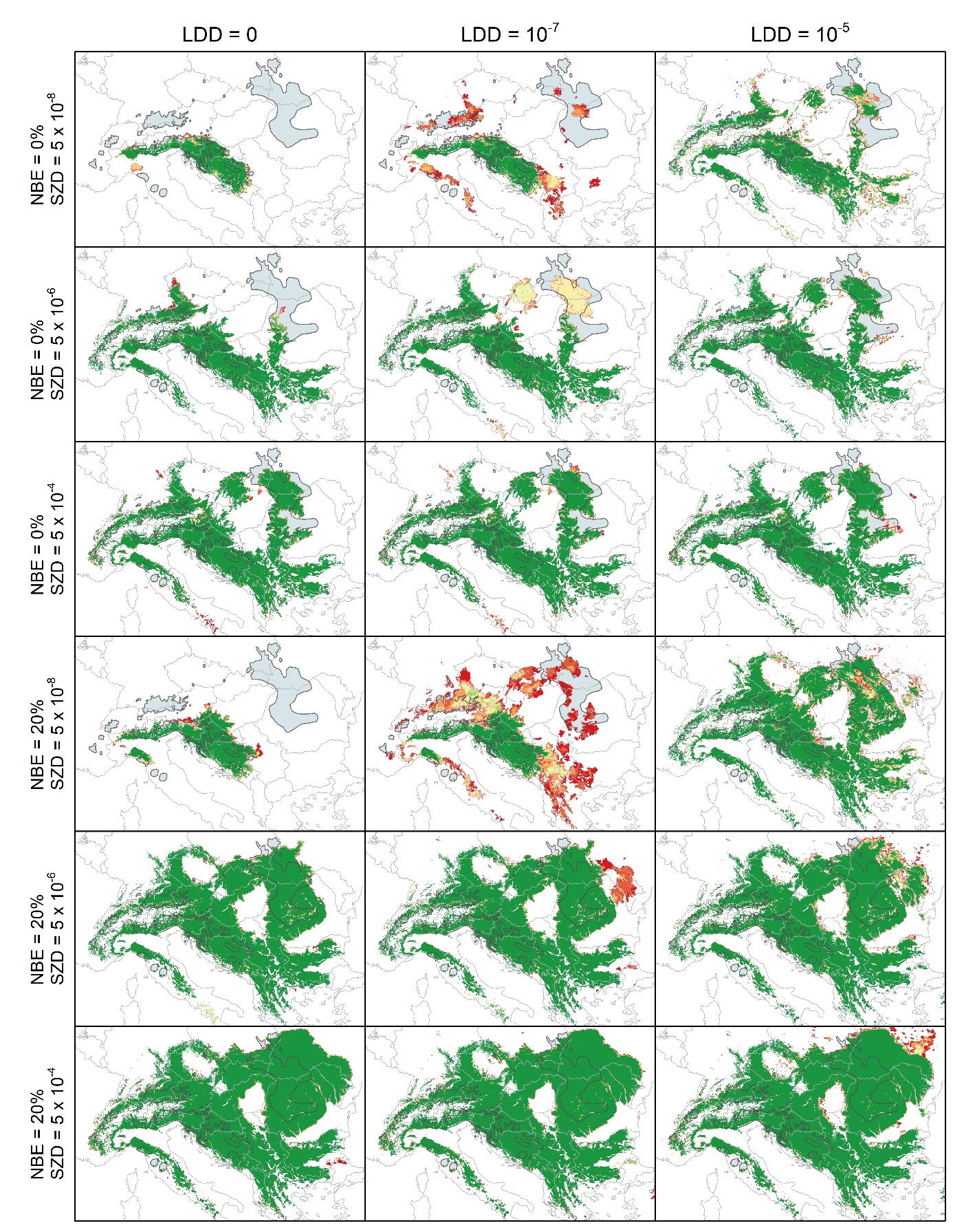
 **Figure S14.** **Af (dem = high).** Predicted range of *Aposeris foetida* in ten replicated model runs using various parameter settings for niche breadth extension (NBE), standard zoochorous dispersal (SZD) and long-distance dispersal (LDD). Demographic rates were assumed to be at the upper end of a plausible range of values. Colours indicate the number of replicates in which a 1 × 1 km cell was occupied, ranging from 1 (dark red) to 10 (dark green). Current species range is shown as blue polygons. For complete simulations, see Videos S24 and S25.


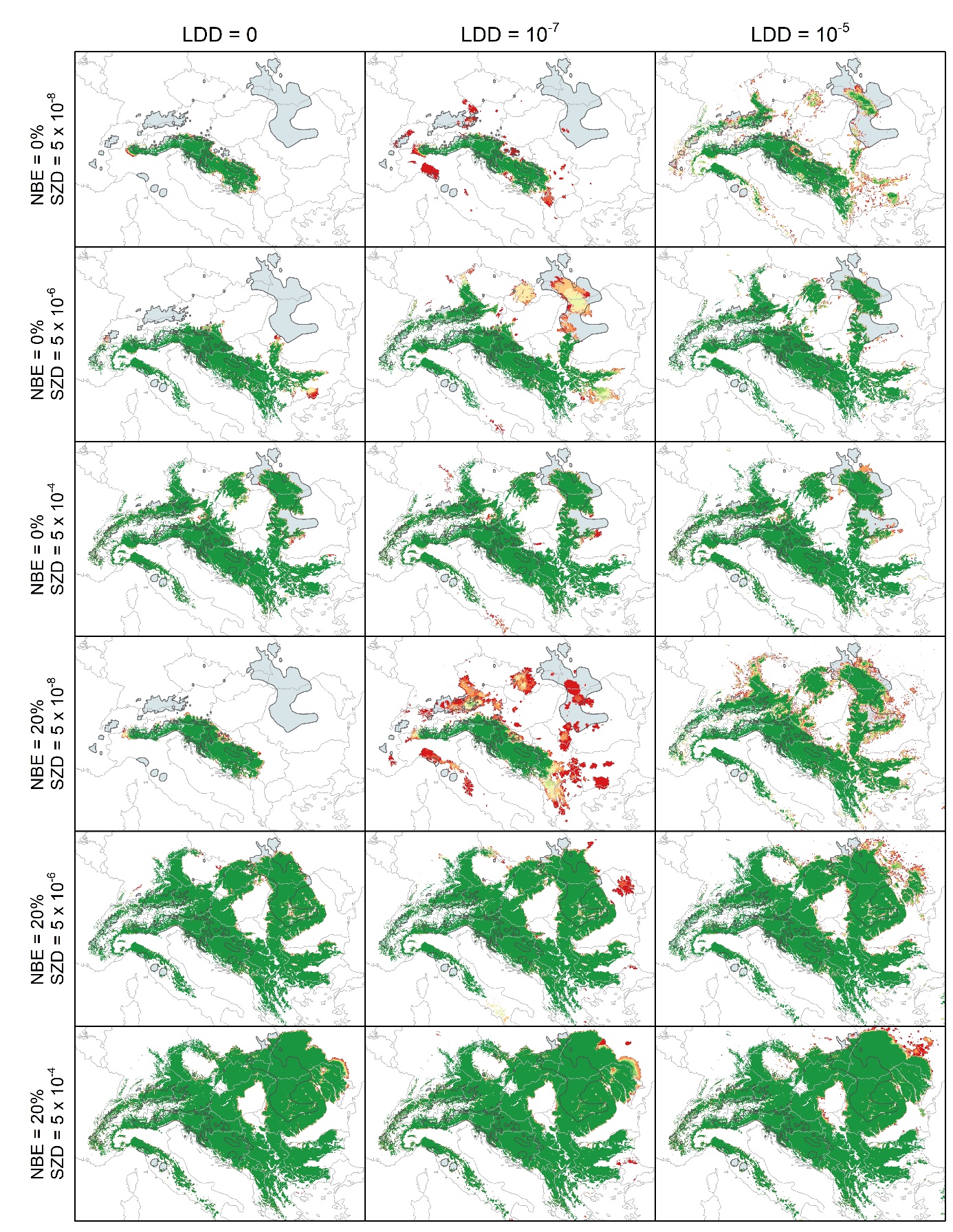


**Figure S15.** **Af (dem = low).** Predicted range of *Aposeris foetida* in ten replicated model runs using various parameter settings for niche breadth extension (NBE), standard zoochorous dispersal (SZD) and long-distance dispersal (LDD). Demographic rates were assumed to be at the lower end of a plausible range of values. Colours indicate the number of replicates in which a 1 × 1 km cell was occupied, ranging from 1 (dark red) to 10 (dark green). Current species range is shown as blue polygons. For complete simulations, see Videos S26 and S27.


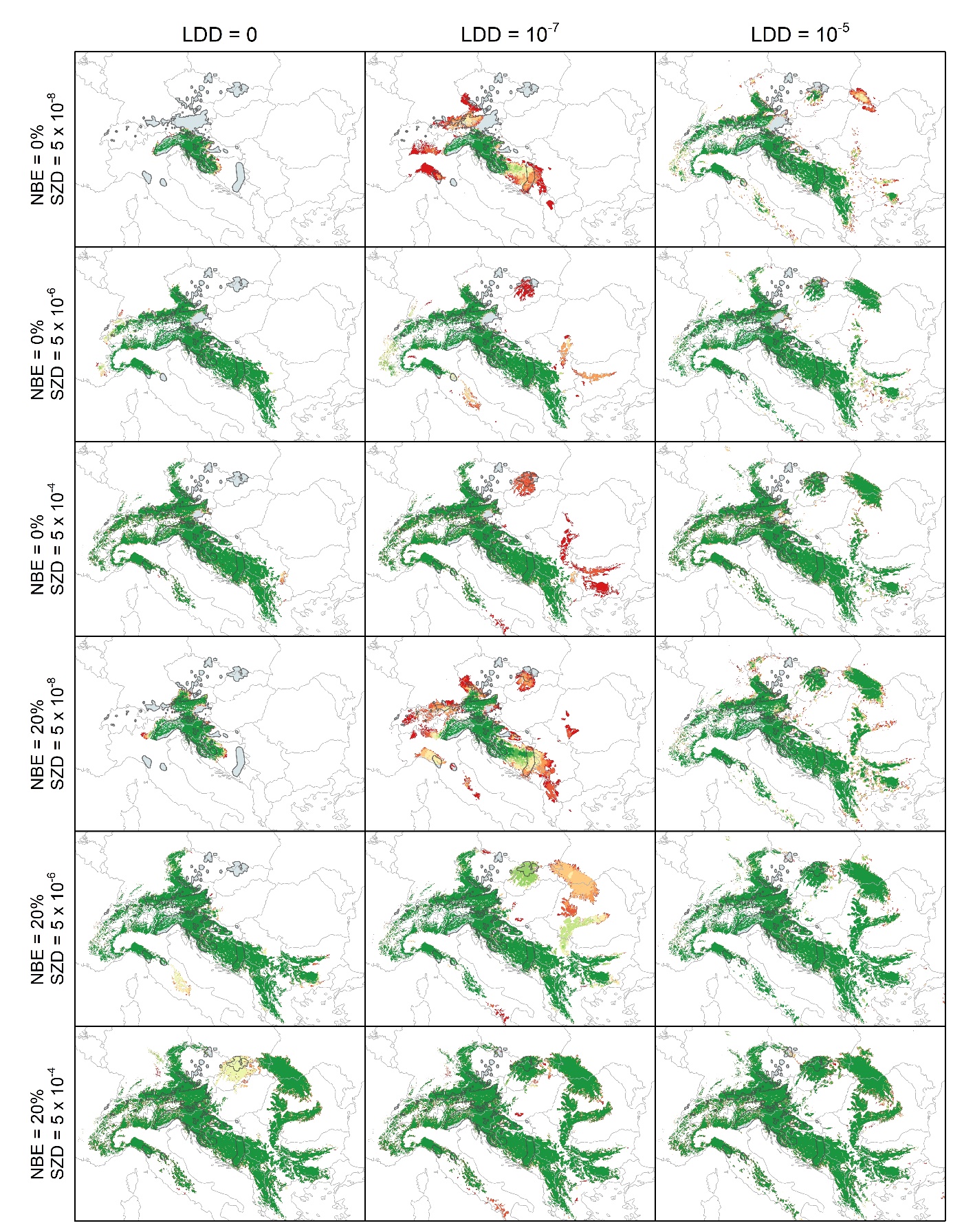


**Figure S16.** **Ct (dem = high).** Predicted range of *Cardamine trifolia* in ten replicated model runs using various parameter settings for niche breadth extension (NBE), standard zoochorous dispersal (SZD) and long-distance dispersal (LDD). Demographic rates were assumed to be at the upper end of a plausible range of values. Colours indicate the number of replicates in which a 1 × 1 km cell was occupied, ranging from 1 (dark red) to 10 (dark green). Current species range is shown as blue polygons. For complete simulations, see Videos S28 and S29.


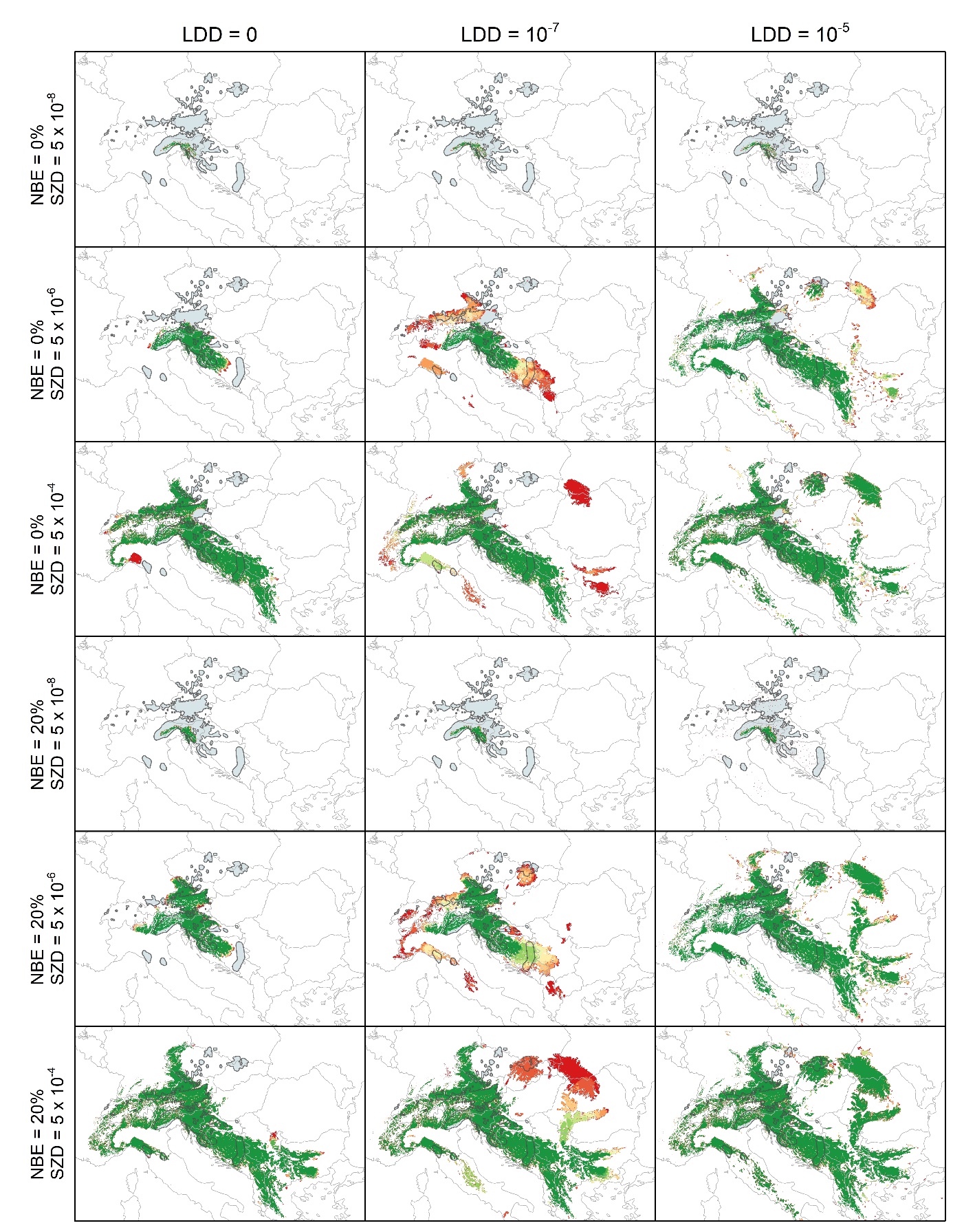


**Figure S17.** **Ct (dem = low).** Predicted range of *Cardamine trifolia* in ten replicated model runs using various parameter settings for niche breadth extension (NBE), standard zoochorous dispersal (SZD) and long-distance dispersal (LDD). Demographic rates were assumed to be at the lower end of a plausible range of values. Colours indicate the number of replicates in which a 1 × 1 km cell was occupied, ranging from 1 (dark red) to 10 (dark green). Current species range is shown as blue polygons. For complete simulations, see Videos S30 and S31.


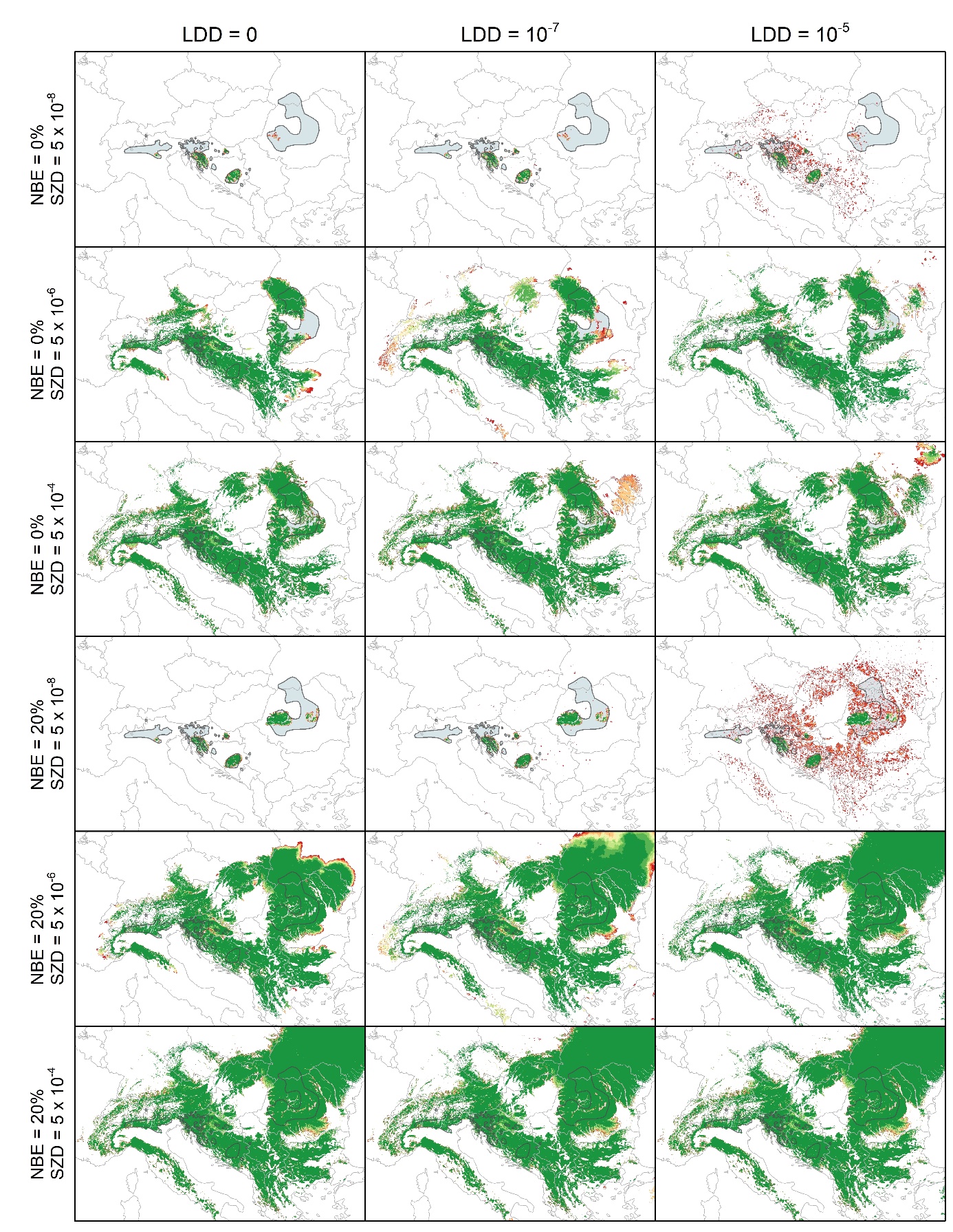


**Figure S18.** **Ec (dem = high).** Predicted range of *Euphorbia carniolica* in ten replicated model runs using various parameter settings for niche breadth extension (NBE), standard zoochorous dispersal (SZD) and long-distance dispersal (LDD). Demographic rates were assumed to be at the upper end of a plausible range of values. Colours indicate the number of replicates in which a 1 × 1 km cell was occupied, ranging from 1 (dark red) to 10 (dark green). Current species range is shown as blue polygons. For complete simulations, see Videos S32 and S33.


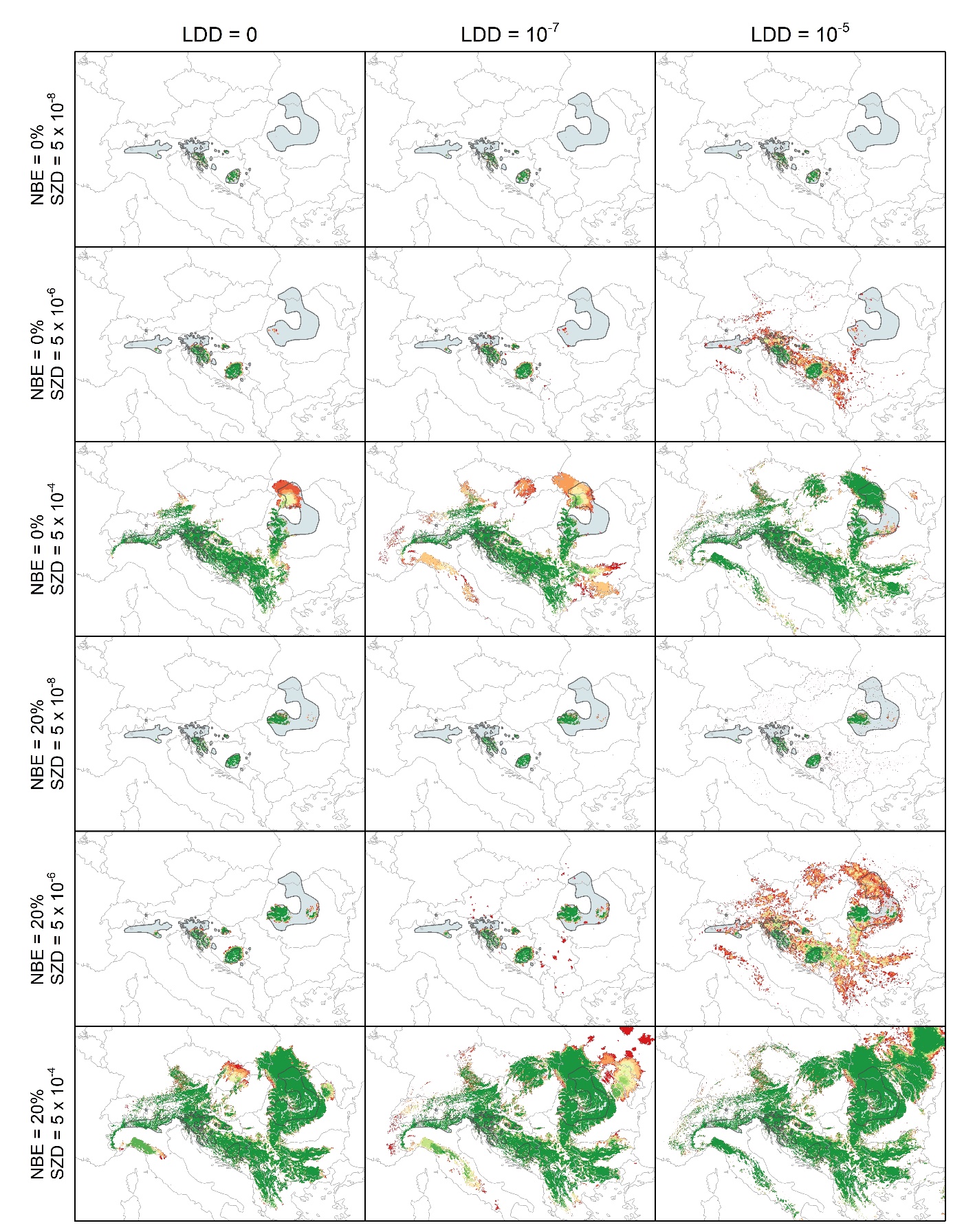


**Figure S19.** **Ec (dem = low).** Predicted range of *Euphorbia carniolica* in ten replicated model runs using various parameter settings for niche breadth extension (NBE), standard zoochorous dispersal (SZD) and long-distance dispersal (LDD). Demographic rates were assumed to be at the lower end of a plausible range of values. Colours indicate the number of replicates in which a 1 × 1 km cell was occupied, ranging from 1 (dark red) to 10 (dark green). Current species range is shown as blue polygons. For complete simulations, see Videos S34 and S35.


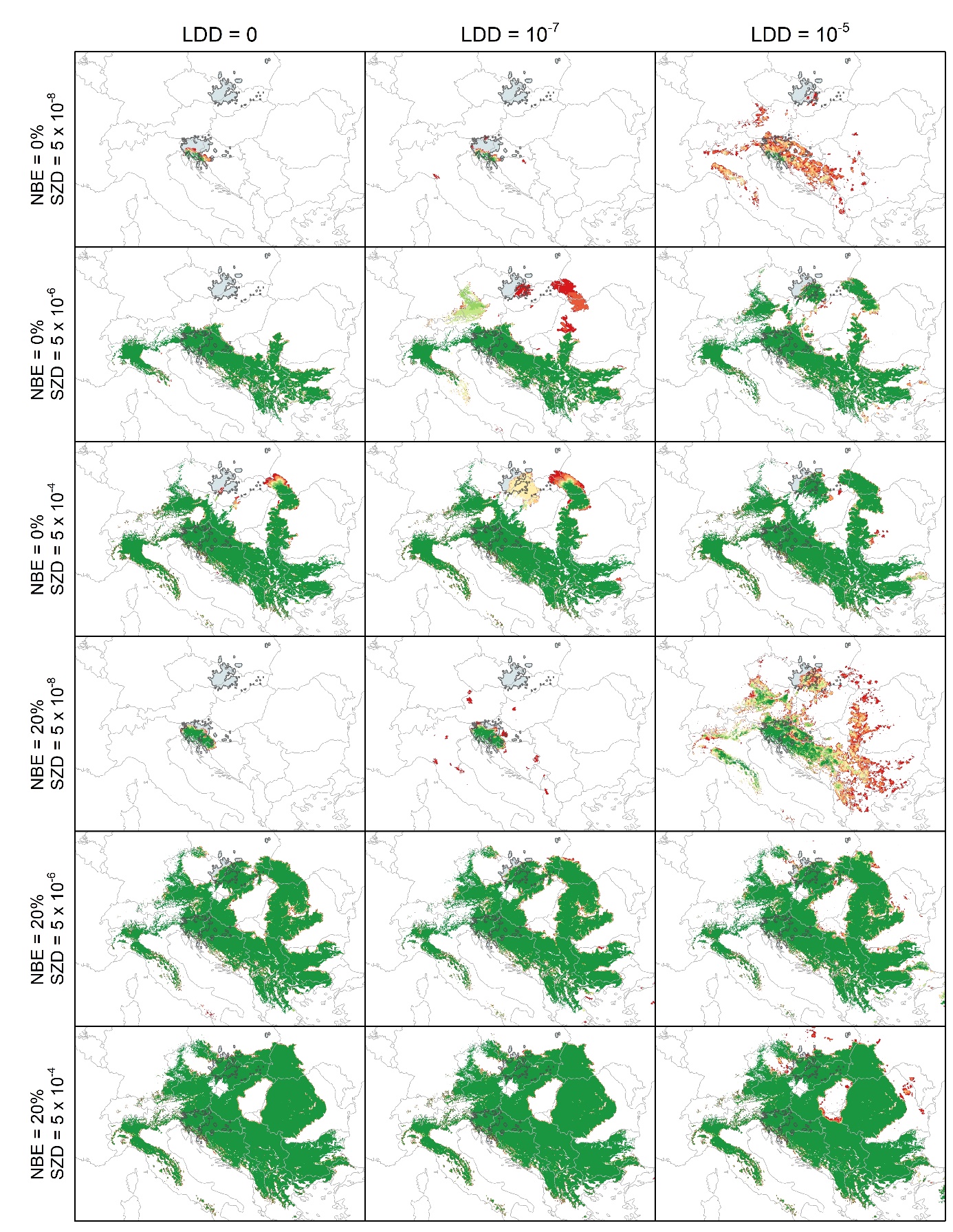


**Figure S20.** **He (dem = high).** Predicted range of *Hacquetia epipactis* in ten replicated model runs using various parameter settings for niche breadth extension (NBE), standard zoochorous dispersal (SZD) and long-distance dispersal (LDD). Demographic rates were assumed to be at the upper end of a plausible range of values. Colours indicate the number of replicates in which a 1 × 1 km cell was occupied, ranging from 1 (dark red) to 10 (dark green). Current species range is shown as blue polygons. For complete simulations, see Videos S36 and S37.


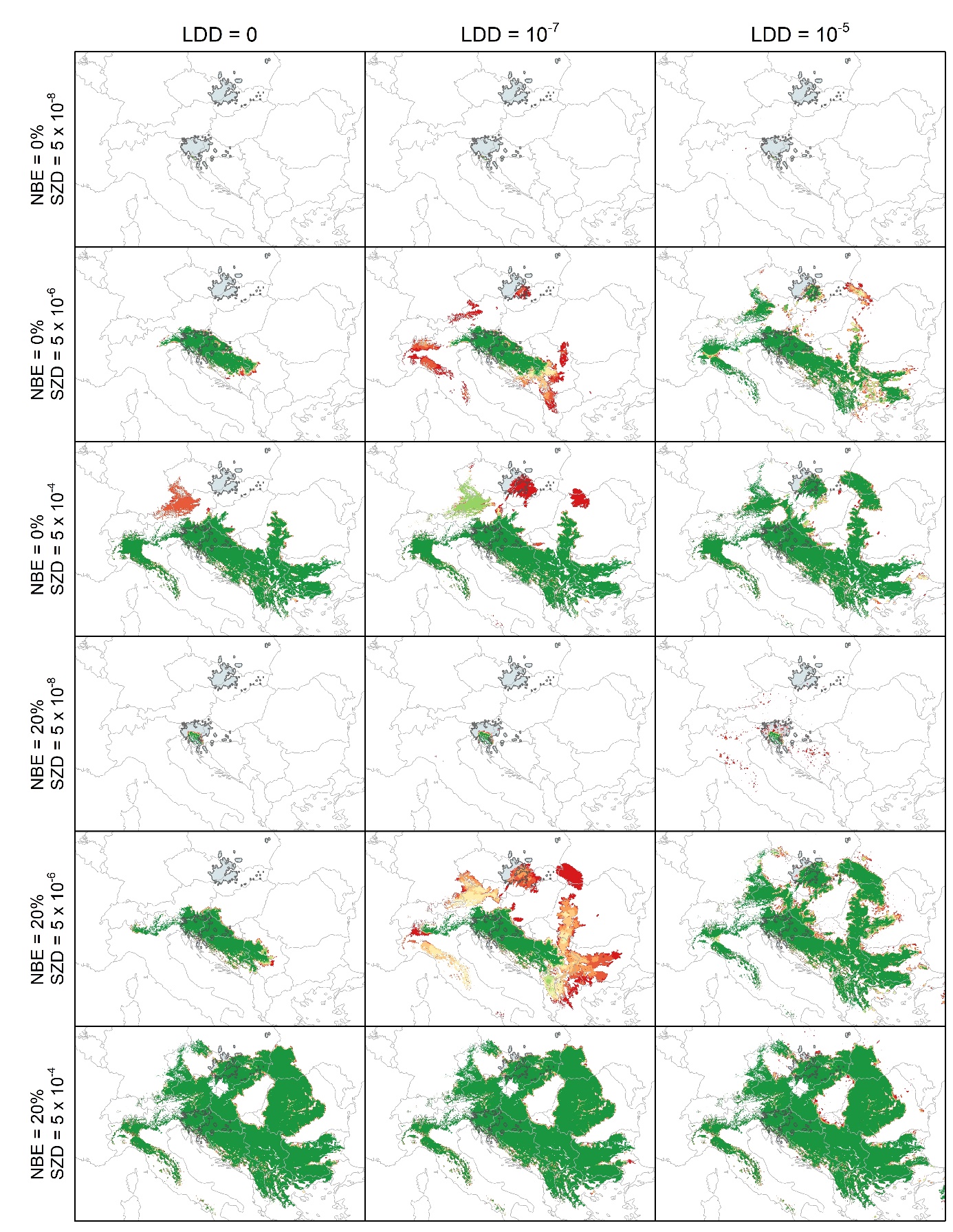


**Figure S21.** **He (dem = low).** Predicted range of *Hacquetia epipactis* in ten replicated model runs using various parameter settings for niche breadth extension (NBE), standard zoochorous dispersal (SZD) and long-distance dispersal (LDD). Demographic rates were assumed to be at the lower end of a plausible range of values. Colours indicate the number of replicates in which a 1 × 1 km cell was occupied, ranging from 1 (dark red) to 10 (dark green). Current species range is shown as blue polygons. For complete simulations, see Videos S38 and S39.


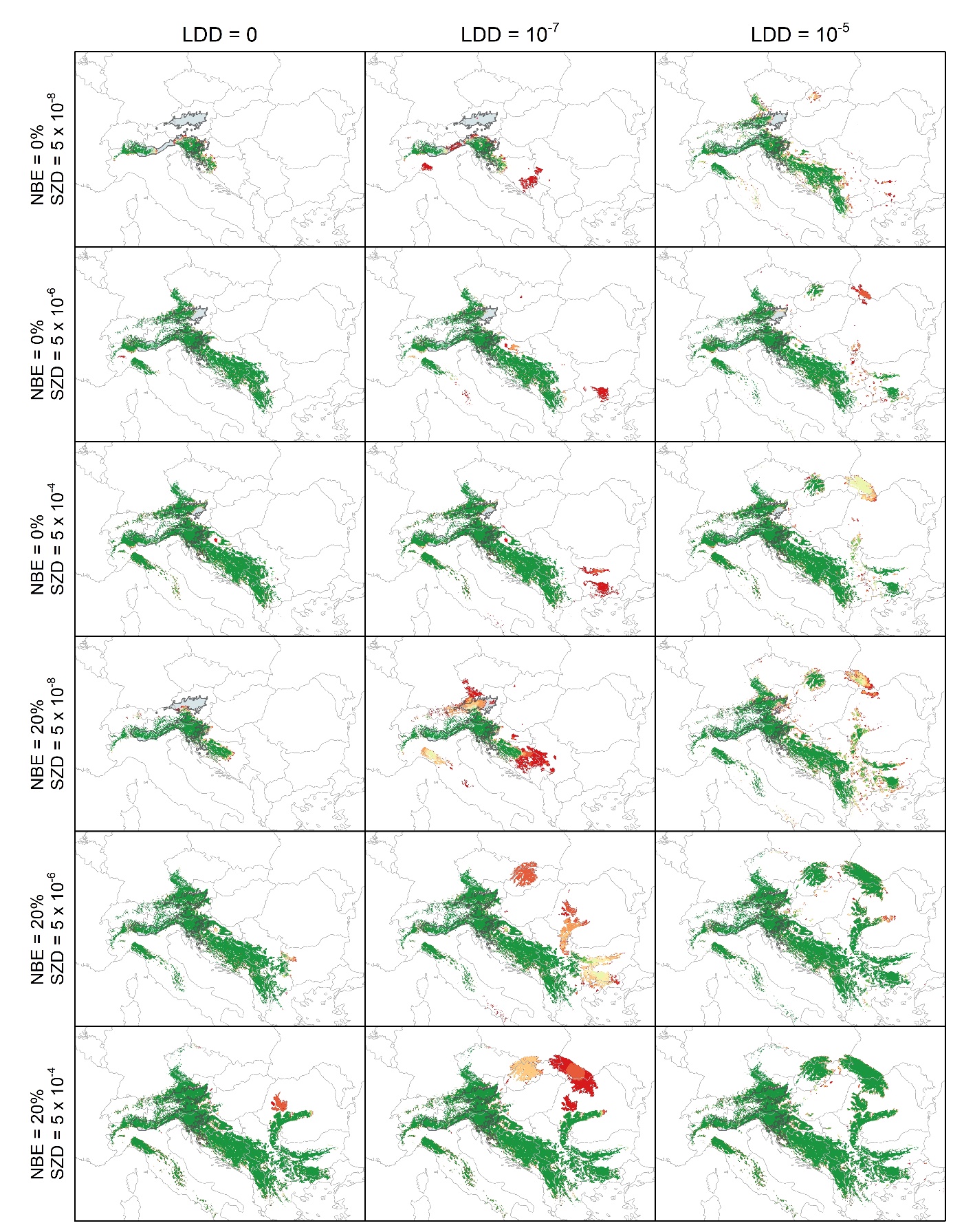


**Figure S22.** **Hn (dem = high).** Predicted range of *Helleborus niger* in ten replicated model runs using various parameter settings for niche breadth extension (NBE), standard zoochorous dispersal (SZD) and long-distance dispersal (LDD). Demographic rates were assumed to be at the upper end of a plausible range of values. Colours indicate the number of replicates in which a 1 × 1 km cell was occupied, ranging from 1 (dark red) to 10 (dark green). Current species range is shown as blue polygons. For complete simulations, see Videos S40 and S41.


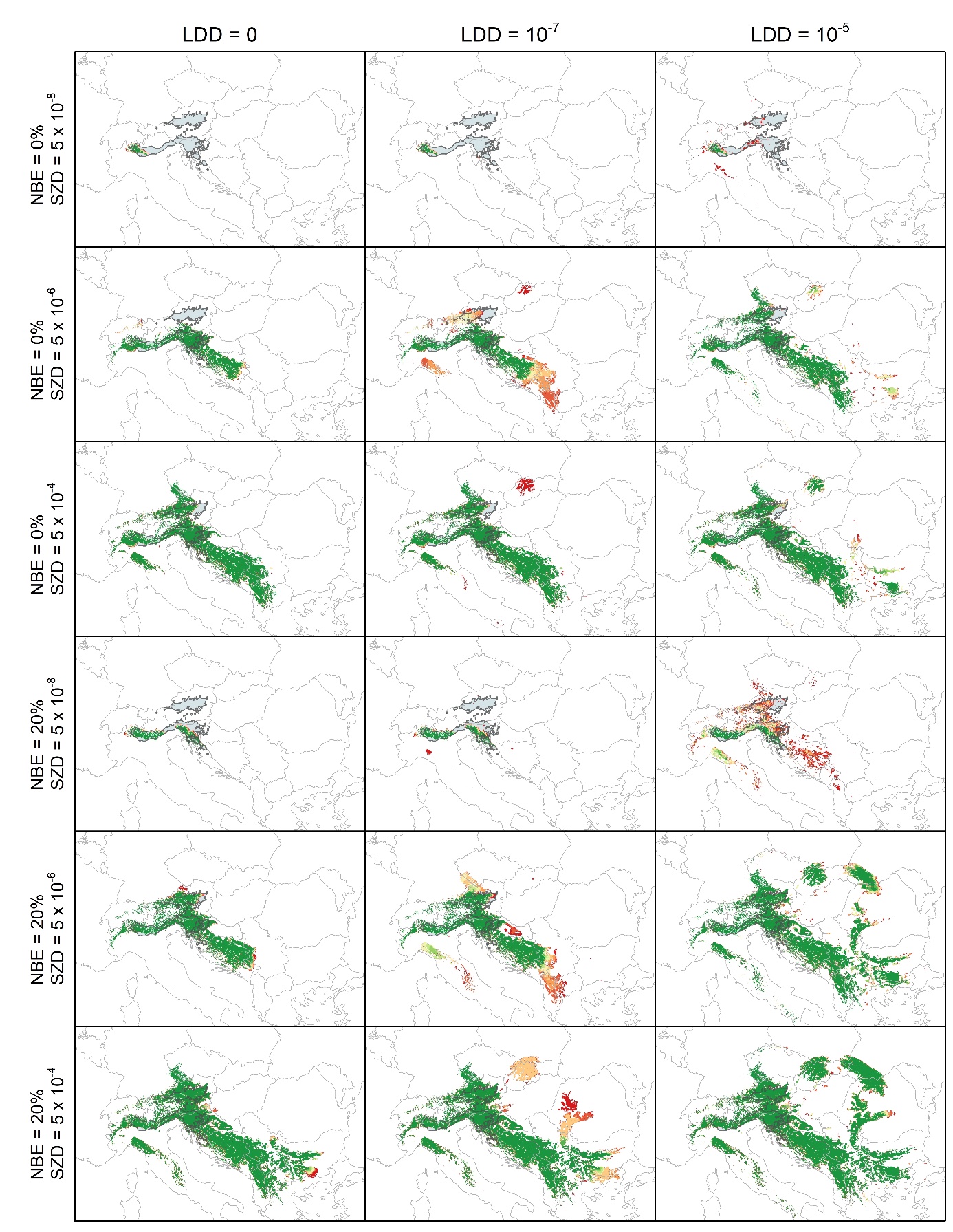


**Figure S23.** **Hn (dem = low).** Predicted range of *Helleborus niger* in ten replicated model runs using various parameter settings for niche breadth extension (NBE), standard zoochorous dispersal (SZD) and long-distance dispersal (LDD). Demographic rates were assumed to be at the lower end of a plausible range of values. Colours indicate the number of replicates in which a 1 × 1 km cell was occupied, ranging from 1 (dark red) to 10 (dark green). Current species range is shown as blue polygons. For complete simulations, see Videos S42 and S43.
